# Impact of Monoclonal Antibody Aggregates on Effector Function Characterization

**DOI:** 10.3390/antib14020031

**Published:** 2025-04-02

**Authors:** Wendy J. Walton, Shousong Jason Zhang, Joseph J. Wilson, Briana N. Harvey, Matthew Clemens, Yingmei Gu

**Affiliations:** 1 Bioproduct Research & Development, Lilly Research Laboratories, Lilly Technology Center North, Indianapolis, IN 46221, USA; 2 Analytical QA, Product Research & Development, Lilly Technology Center North, Indianapolis, IN 46221, USA

**Keywords:** monoclonal antibody, aggregation, Fc receptor, forced degradation, binding, surface plasmon resonance

## Abstract

Background/Objectives: Monoclonal antibodies have successfully been used for a variety of indications. Many therapeutic antibodies are IgG1 and elicit effector functions as part of their mechanism of action. It is well known that aggregate levels should be controlled for therapeutic antibodies. Although there are several reports describing the impact of antibody aggregates on FcγR binding, most of these have been performed with surface plasmon resonance in an avidity-based format. What is less well known is which Fcγ receptor is most impacted by antibody aggregation and how antibody aggregates impact binding to Fcγ receptors in solution-based formats and in cell-based assays. Methods: An effector-competent IgG1 (mAb1) was forcibly degraded and fractionated by size exclusion chromatography to enrich for aggregates. The fractions were examined for FcγR binding by SPR with different formats and in solution. The fractions were also analyzed with cell-based FcγR reporter assays. Results: All Fcγ receptors displayed increased binding to enriched mAb1 aggregates in the avidity-based SPR methods and in solution, with FcγRIIa impacted the most. When examined with an antibody-down SPR format that is not usually susceptible to avidity, FcγRIIa did not show increased binding with mAb1 aggregation. Although activity for mAb1 aggregates increased slightly in an FcγRIIa cell-based reporter assay, it decreased in the FcγRIIIa reporter assay (most likely due to differences in fucosylation from the reference standard). Conclusions: Monoclonal antibody aggregation can impact FcγR binding for avidity-based binding formats. Even at low levels of antibody aggregation, FcγRII binding increases substantially.

## 1. Introduction

At least one hundred therapeutic monoclonal antibodies (mAbs) have been approved by the FDA for various indications [1]. Monoclonal antibodies can elicit (or block) responses by target-specific binding through their antigen-binding fragment (Fab), as well as induce immune responses via interactions of the crystallizable fragment (Fc) with Fcγ receptors (FcγR) [2]. The Fc region of an IgG can bind to various FcγRs on immune effector cells to induce antibody-dependent cellular cytotoxicity (ADCC) or antibody-dependent cellular phagocytosis (ADCP) by monocytes/macrophages [3]. C1q binding to the Fc can also promote cell death via complement-dependent cytotoxicity (CDC), in which mAb binding to target cells results in the activation of the complement cascade via the binding of C1q to the mAb Fc region [4]. Among the IgG-binding FcγRs, the human Fc gamma receptor (FcγR) has three main classes, FcγRI, FcγRII, and FcγRIII, which are expressed on different immune cells. FcγRI is the only high-affinity FcγR, and is expressed on monocytes, macrophages, neutrophils, mast cells, eosinophils, and dendritic cells [5,6,7]. FcγRII includes three different isoforms: FcγRIIa, FcγRIIb, and FcγRIIc [3,5,8]. FcγRIIa has two allelic variants, which correspond to changes in the amino acid at position 131 (FcγRIIa-131Arg and FcγRIIa-131His). The FcγRIIa-131H allelic variant has a greater affinity for IgG1 and IgG2 [5,9]. FcγRIIa is expressed by monocytes, macrophages, neutrophils, platelets, mast cells, eosinophils, dendritic cells, and T cells [5,6,7]. FcγRIII consists of FcγRIIIa and FcγRIIIb isoforms. FcγRIIIa has two different allelic variants: a higher-affinity allelic variant (FcγRIIIa-158Val) and a lower-affinity allelic variant (FcγRIIIa-158Phe). FcγRIIIa is expressed from monocytes, macrophages, neutrophils, natural killer cells, and T cells [5,6,7].

For desired potency, efficacy, and safety, mAb activity and stability in the drug product must be maintained throughout production, processing, and storage. Increased aggregation due to mechanical, thermal, or chemical stress during manufacturing and storage is a critical quality attribute that can alter the immunogenicity of mAbs by inducing an inflammatory response and FcγR-mediated innate cell activation [10,11]. The aggregation of protein therapeutics can decrease their efficacy by reducing the effective concentration, as well as promoting the induction of anti-drug antibody (ADA) production [10,12]. ADA production could potentially lead to a series of complications [10]. Protein aggregates can range considerably in size, from dimers (nm), oligomers (nm–0.1 μm), and linear or amorphous sub-visible (0.1–100 μm) aggregates to visible (>100 μm) particulates [13]. Methods commonly used to analyze and monitor mAb size variants/higher order structures include size exclusion chromatography (SEC), analytical ultracentrifugation, light scattering, micro-flow imaging, and native gel electrophoresis [14]. Forced or induced degradation by different stressors such as temperature, pH, and oxidation are frequently used during therapeutic mAb development to understand degradation pathways and critical quality attributes, as well as to assess analytical method capability and suitability [15,16,17].

There have been several reports of aggregated IgG increasing FcγR-mediated activity. Early reports were based on observations of aggregated immunoglobulins displaying enhanced binding and activation of macrophages [18,19], and later with PBMCs [20,21,22,23] and monocytes [23], where mAb or IVIG aggregates induced an immune response measured by cytokine release. FcγR blocking studies showed Fcγ receptors to be partly involved in promoting cytokine release [20,23]. Tada et al. demonstrated aggregate-dependent activity in FcγRI-, FcγRIIa-, and FcγRIIIa-expressing reporter cells for two different IgG1 mAbs. Aggregates smaller than 1 μm directly activated FcγRIIa and FcγRIIIa reporter cells [24]. Utilizing IgG–antigen immune complexes, Lux et al. demonstrated a size-dependent increase in binding to FcγR-expressing cells for the low-affinity receptors, but not FcγRI [25]. IgG aggregates increase FcγR binding and retention when measured by SPR [26,27,28,29] or SPRi [30] in an avidity-driven manner (with FcγR on the chip surface).

The impact of IgG aggregates on both FcγR binding and cell-based activity have been evaluated in only a few cases, and all but one [31] utilized effector cells expressing multiple Fcγ receptors. One study found varying impact to ADCC activity and FcγRIIIa binding affinity by SPR with a rituximab biosimilar exposed to different forced degradation conditions [32]. Given the amount of additional modifications (e.g., fragmentation, deamidation, etc.) produced by the forced degradation conditions, it is difficult to interpret the impact of aggregation alone. Lopez et al. showed that IgGs with higher levels of aggregates led not only to increased FcγRII and FcγRIII binding, but also increased ADCP activity with THP-1 cells [29]. Although conclusively showing a correlation of increased FcγR activity with low pH exposure and increased aggregation, the study focused on the impact of different purification methods for IgG from plasma, and as samples were mixtures, it was not possible to fully characterize individual IgGs. Nagashima et al. reported that constructs containing Fc tandem repeats led to increased binding to the low-affinity Fcγ receptors by ELISA, as well as increased activity in ADCC with PBMCs [33] and ADCP with THP-1 cells [34]. Bajardi-Taccioli et al. found aggregate-driven increases in FcγRIIa binding in an AlphaScreen assay, but similar increases were missing in an ADCP assay utilizing FcγRIIa-expressing effector cells [31].

In this study, we examined the impact of aggregates for an effector-competent IgG1 (designated mAb1) on a variety of functional assays, with sample results calculated relative to a reference standard. We show that subtle changes in mAb1 aggregation can lead to a dramatic increase in FcγR binding, with avidity-based binding assays that differ in behavior to the reference standard, but do not show a similar impact in the cell-based FcγR reporter assays.

## 2. Materials and Methods

### 2.1. Materials

Effector-competent IgG1 mAb1 expressed from Chinese hamster ovary cells in histidine buffer containing NaCl, sucrose, and polysorbate 80, pH 6 at 35 and 150 mg/mL was provided by Eli Lilly and Company, Indianapolis, IN, USA. mAb1 concentrations were obtained by UV analysis at 280 nm, utilizing the mAb1 extinction coefficient. One mAb1 reference standard (Ref) at 9.6 mg/mL was used throughout the entire study.

### 2.2. Forced Degradation Study

mAb1 samples at 35 mg/mL were exposed to relevant stress conditions, such as pH (±2 pH unit around the target pH 6.0), hydrogen peroxide (1, 10 ppm), iron (0.5, 20 ppm), copper (20 ppm), and 200 mM glucose as indicated in Table 2, in suitable glass containers stored at 40 °C for 28 days.

### 2.3. Photostability Studies

Samples at 150 mg/mL (Batches A and C) and 35 mg/mL (Batch B) were light-stressed in borosilicate glass vials according to ICH Q1B guidelines [35]. Borosilicate glass vials (20 mL) covered with metal foil served as dark controls. Batches A and B were exposed to visible light at 1.2 M lux hours/200 W hours/m^2^ UV light at 20 °C. Batch C was exposed to 110% of ICH Q1B guidelines at 1.32 M lux hours/220 W hours/m^2^ UC light at 20 °C.

### 2.4. Size Exclusion Chromatography (SEC)

#### 2.4.1. Analytical SEC

Samples were diluted to 1 mg/mL with mobile phase buffer (50 mM NaH_2_PO_4_, 300 mM NaCl, pH 7.0) prior to analysis. SEC-HPLC was performed with an Agilent HPLC system and Tosoh Bioscience TSK-Gel G3000SWxl, 7.8 × 300 mm, 5 µm particles (Tosoh, Tokyo, Japan) with isocratic elution. The chromatographic profile was monitored using UV detection at 214 nm at a 0.5 mL/min flow rate with a column temperature of 25 °C, injection volume of 10 µL, and run time of 30 min. The sum for all peaks that eluted prior to the monomer was reported as total aggregates (%) and the sum areas for all peaks that eluted after the monomer (excluding buffer peaks) were reported as total fragments (%).

#### 2.4.2. Preparative SEC

Preparative SEC purification was performed on the mAb1 batch C exposed to 110% of ICH Q1B guidelines. A Superdex 200 50/60 (1140 mL) column was equilibrated with Histidine/NaCl/Sucrose buffer using an ÄKTA Avant 150 system (Cytiva life sciences, Marlborough, MA, USA) at a flow rate of 8 mL/min. A fraction collector was utilized to collect 13 mL fractions based on a UV 280 nm signal. Pooled fractions were filtered with a 0.22 µm filter, stored at 2–8 °C, and analyzed by analytical SEC to determine the percentages of monomer, dimer, and higher oligomeric species in each fraction. The mAb concentrations of each fraction were determined by UV absorbance at 280 nm.

### 2.5. Capillary Gel Electrophoresis–Sodium Dodecyl Sulfate (CE-SDS)

CE-SDS was performed under both reducing and non-reducing conditions for the analysis of purity/impurities. A Beckman Coulter PA 800 capillary electrophoresis system and IgG Purity and Heterogeneity Kit (Sciex) were used, along with a 10.2 cm (for non-reduced) or 20 cm (for reduced) long bare silica capillary (from the sample introduction inlet to detection window) and 100 × 200 µm aperture. For reduced CE-SDS, samples were diluted to 2 mg/mL in 100 mM Tris-HCl, pH 9.0, containing 1% SDS, reduced using 2% β-mercaptoethanol, and subjected to electrophoresis under reducing conditions. For non-reduced CE-SDS, samples were diluted to 1 mg/mL in 100 mM Tris-HCl, pH 6.8, with 1% SDS, alkylated using 25 mM iodoacetamide, and subjected to electrophoresis under non-reducing conditions.

### 2.6. Mass Spectrometry

For characterization by mass spectrometry, mAb1 samples were prepared and analyzed mostly according to Cain et al. [36]. Samples were digested with Lys-C and Trypsin after denaturation with 6 guanidine HCl, reduction with DTT, and alkylation with iodoacetamide. Forcibly degraded samples and photostability samples from mAb1 batches A and B were quenched with an acetic acid solution containing methionine before LC/MS analysis. The digests were analyzed on a Thermo Q Executive Plus mass spectrometer after separation with a Waters Acquity Peptide UPLC BEH 300 C18, 2.1 × 150 mm, 1.7 µm particle size column. Separations were achieved using a water/acetonitrile gradient with TFA in 92 minutes. The batch C photostability sample and isolated peaks were mixed with 66% TFA in water before LC/MS/MS analysis. The digests were analyzed on a Thermo Exploris 480 mass spectrometer after separation, as described above, but with a 60 min gradient. Data were analyzed using Biopharma Finder to determine the relative quantities of peptide post-translational modifications. The default component ions (software selected) were used in the percent abundance calculations without any customization of the calculations.

### 2.7. SPR Binding Assays with Ref Standard Curves

The neonatal Fc receptor (FcRn) binding assay was performed with a Biacore T200, as previously reported [37]. The FcγRIIa, FcγRIIIa, and antigen assays were also completed on a Biacore T200, in a similar manner to the FcRn assay, with differences as outlined below. One downstream flow cell of an SA Sensor Chip (Cytiva, Marlborough, MA USA) was immobilized with biotinylated Avi-tag FcγRIIa (131R, ACROS Biosystems, Beijing, China or Sino Biological, Beijing, China) or Avi-tag FcγRIIIa (158V, Sino Biological) at levels between 1300 and 2400 resonance units (RU), according to the instructions recommend for the SA chip. One downstream flow cell of a CM5 Sensor Chip (Cytiva) was immobilized with FcγRIIIa (158Val, ACROS Biosystems or in-house [38]) or mAb1 antigen at levels of ~2200 RU or ~15000 RU, respectively, utilizing an amine coupling kit (Cytiva). Blank flow cells were prepared upstream of FcγRIIa and FcγRIIIa chips. Standard curves were prepared, with a total of nine different concentrations serially diluted 1.5-fold using the mAb1 Ref (150–6 µg/mL for FcγRIIa and FcγRIIIa assays with SA chip; 460–18 µg/mL for FcγRIIIa with CM5 chip; and 6–0.2 µg/mL for antigen with CM5 chip). The mAb samples were tested at 5 concentrations from 70 to 14 µg/mL (FcγRIIa and FcγRIIIa with SA chip), 200–40 µg/mL (FcγRIIIa with CM5 chip), and 3.2–0.6 µg/mL (antigen binding with CM5 chip), as determined from absorbance at 280 nm. Samples that had RU values above the calibration curve were tested again with a more dilute sample. HBS-N (Cytiva) was utilized as the running buffer and sample dilution buffer for the FcγRIIIa assays. The surface was regenerated for 3 s with 12 mM NaOH (FcγRIIa on SA chip), 10 s with 0.1 M Na_2_CO_3_/1M NaCl (FcγRIIIa on SA chip), 4 s with 10 mM Na_2_CO_3_/1 M NaCl (FcγRIIIa on CM5 chip), and 25 s with 4 M MgCl_2_ (antigen binding with CM5) at a flow rate of 75 µL/min. Reference flow cells upstream of the active flow cell were utilized for the Fc receptor binding assays with reference-subtracted baseline-relative RU captured 7 s before the end of the injection. For the antigen binding assay, baseline-relative RU 10 s after the end of the injection was utilized. mAb1 was injected at 30 µL/min. The RU from the mAb1 Ref standard curves was fitted with a 4-parameter model to obtain a calibration curve. Standard curves at the beginning and end of each run were utilized for the implementation of calibration trend sample analysis. For each sample, concentrations calculated from the Ref calibration trend and multiplied by the sample dilution were then divided by the sample UV concentration to obtain the proportion of sample capable of receptor binding.

### 2.8. Kinetics, Affinity, and Direct Binding SPR Assays

For the SPR “Fc-receptor-down” affinity assay (avidity method), 10 RU of biotinylated FcγRIIa (131R, Sino Biologicals) was immobilized onto a Streptavidin (SA) Sensor Chip (Cytiva) flow cell. Ref and isolated fraction 2 (Peak 2) were injected at concentrations between 1000 and 0.15 nM for 300 s at 30 µL/min for both the active flow cell and an upstream blank flow cell, and allowed to dissociate for 600 s. The surface was regenerated at a flow rate of 75 µL/min for 3 s with 12 mM NaOH. Reference- and buffer-subtracted responses were fitted to a steady-state 1:1 binding model for Ref.

For the SPR “mAb-down” affinity assay (non-avidity method), a CM5 chip immobilized with ~15,000 RU antigen via amine coupling on the reference and active flow cell was utilized to capture 80–200 RU of mAb1 on the active flow cell. FcγRIIa 131R (Eli Lilly and Company [38]) was injected at concentrations between 6000 and 25 nM for 100 s at 30 µL/min over both flow cells, and allowed to dissociate for 200 s. Reference- and buffer-subtracted responses were fitted with the evaluation software to determine steady-state affinity for both Ref and Peak 2. Ref and Peak 2 were analyzed with both formats during three separate runs. For FcγRIII 158V, approximately 175 RU of Ref and Peak 4 were captured by antigen and FcγRIII 158V was injected at concentrations between 3000 and 1 nM for 200 s at 30 µL/min over both flow cells and allowed to dissociate for 400 s. Reference-subtracted binding responses for FcγRIIIa binding at 7 s before the end of the injection were plotted for each concentration.

To measure SPR responses during binding and dissociation, FcγRs were covalently immobilized to CM5 flow cells via amine coupling. Samples (0.6 µM) were injected at 30 µL/min for 200 s four times. Reference flow cell-subtracted responses 7 s before the end of the injection (binding response) and 10 s post injection (response during dissociation) were divided by the average responses for the Ref.

All SPR assays utilized a Biacore T200 (Cytiva) utilizing method builders under the control of Biacore T200 Control software v 2.0, with high-performance sample injections, one conditioning step, 3 startup runs, analysis performed at 25 °C, and the sample compartment set at 4 °C, and were analyzed with Biacore T200 Evaluation Software v 3.0. Unless otherwise indicated, all concentrations were injected in duplicate and HBS-EP (Cytiva) was utilized as a running buffer and sample dilution buffer.

### 2.9. Solution-Based Competitive Fc Receptor Binding Assays

Competitive no-wash binding assays were performed in non-binding white plates (Corning, Corning, NY, USA) with the Lumit Binding Immunoassays (Promega, Madison, WI, USA) for FcγRI, FcγRIIa (131H, 131R), and FcγRIIIa (158V, 158F) according to the manufacturer’s instructions. Plates were read on a luminometer utilizing SoftMax Pro v. 7 (Molecular Devices, San Jose, CA, USA). Each sample was measured in duplicate on the same plate, and average responses were fitted to a 4-parameter logistic nonlinear regression model. Sample potencies were calculated relative to Ref utilizing a constrained model [39] with SoftMax Pro v. 7. The isolated fractions 1–3 and photostability sample did not meet the requirements for similarity to the Ref in the FcγRIIa competitive binding assays. A non-constant mean was utilized to calculate the relative potency [40] for these samples with SoftMax Pro v. 7. Samples were measured on two different plates, in duplicate on each plate, for each receptor, and an average relative potency was obtained for each sample. Average responses were also plotted against the mAb1 concentration with GraphPad Prism 10.1.1.

### 2.10. FcγRIIa and FcγRIIIa Reporter Gene Assays

#### 2.10.1. FcγRIIa Assay

After adding ~13,000/well antigen-expressing target cells (or 25 µL media) to white cell culture-treated 96-well plates (Corning) and incubating the plates at 37 °C, 5% CO_2_ for 15 min, dilutions of mAb1 at plated concentrations from 200 µg/mL to 64 ng/mL were added and plates were placed at 37 °C, 5% CO_2_ for another 15 min. Thaw-and-use effector cells (FcγRIIa 131H) were prepared according to the vendor (Promega), with 77,500/well for effector-to-target-cell ratios between 5.1 and 5.8. After ~16 h, luciferase activity was measured on a luminometer with Bright-Glo^TM^ (Promega). Each sample was measured in triplicate on the same plate and the median responses were plotted against the mAb1 concentration with GraphPad Prism 10.1.1. Additionally, as the Ref did not show a dose-dependent response, the responses for the 110% ICH and isolated fractions for the 200 µg/mL dose were divided by the average response for the Ref at 200 µg/mL on each plate to obtain a relative response for each sample replicate. Each sample was measured with a minimum of two replicates and averaged.

#### 2.10.2. FcγRIIIa Assay

The FcγRIIIa reporter gene assay with target cells was performed based on a previous study [41]. After adding ~13,000/well antigen-expressing target cells to white-cell-culture-treated 96-well plates (Corning), serial dilutions of mAb1 at final concentrations of 5000–1 ng/mL were added and plates were placed at 37 °C, 5% CO_2_ for 1 h. Antibody and target cells were prepared in serum-free media. Thaw-and-use effector cells (FcγRIIIa 158V) were prepared according to the vendor (Promega), with ~76,000 cells/well for effector/target cell ratios between 5.3 and 6.6. After ~20 h at 37 °C, 5% CO_2_ Bio-Glo^TM^ (Promega) was utilized to measure luciferase activity on a luminometer (Molecular Devices). Each sample was measured in triplicate on the same plate and the median responses were fitted to a 4-parameter logistic nonlinear regression model with SoftMax Pro v. 7 (Molecular Devices). Sample potencies were calculated relative to mAb1 Ref. As the samples did not meet the requirements for similarity to Ref, most likely due to differences in fucosylation, a non-constant mean was utilized to calculate the relative potency [40] for the samples utilizing SoftMax Pro v. 7. Each sample was measured on three plates, with the geometric mean and confidence intervals of the relative potencies calculated with Excel. Median responses were also plotted against the mAb1 concentration with GraphPad Prism v. 10.

For plates comparing ± target cells, the methods above were modified by utilizing mAb1 serial dilutions at plated concentrations from 200 µg/mL to 64 ng/mL and adding 25 µL media for wells without target cells. Since the Ref did not show a dose-dependent response, the responses for the 110% ICH and isolated fractions for the 200 µg/mL dose were divided by the average response for the Ref at 200 µg/mL on each plate to obtain a relative response for each sample replicate. Each sample was measured in duplicate per plate, and the relative responses obtained for each plate were averaged for the three plates.

### 2.11. Statistical Data Analysis

Regression analysis was performed with JMP statistical software v. 17 for correlation analysis. In all cases, *p*-values < 0.05 were considered statistically significant.

## 3. Results

### 3.1. Characterization of Photostability and Forcibly Degraded mAb

IgG1 (mAb1), exposed to 100% photostability conditions per ICH Q1B, was analyzed for FcγRIIa (131R), FcγRIIIa (158V), and FcRn binding by surface plasmon resonance (SPR) to determine the impact of light stress on FcγR binding. All results were calculated based on a reference standard curve. Compared to the dark control, the percentage of FcγRIIa binding was greater in the light-exposed samples (Table 1) and well outside the variability observed with this FcγRIIa binding method (3.7% RSD intermediate precision with ranges between 0.9 and 1.1 relative binding).

The light-exposed samples had higher levels of aggregates compared to the dark control samples, as determined by size exclusion chromatography (SEC) and capillary electrophoresis–sodium dodecyl sulfate (CE-SDS) under reduced and non-reduced conditions. mAb1 was treated with 2% β-mercaptoethanol before analysis by reduced CE-SDS, and thus the aggregates detected by reduced CE-SDS were designated as non-reducible aggregates. The slight increase in FcγRIIIa binding for the 150 mg/mL light-exposed material compared to the dark control could be related to increased aggregation levels compared to the reference standard (Ref). The increase in aggregation for batch A (150 mg/mL) compared to batch B (35 mg/mL) aligns with results seen by others, where aggregation propensity increases with mAb concentration [42]. There was a slight decrease in FcRn binding for the light-exposed samples due to more than 20% oxidation in corresponding Met252 and Met428 (based on EU numbering). These sites are known to impact FcRn binding [37,43,44,45]. Additional samples were forcibly degraded to examine the impact on FcγRIIa binding (Table 2).

**Figure 1:**
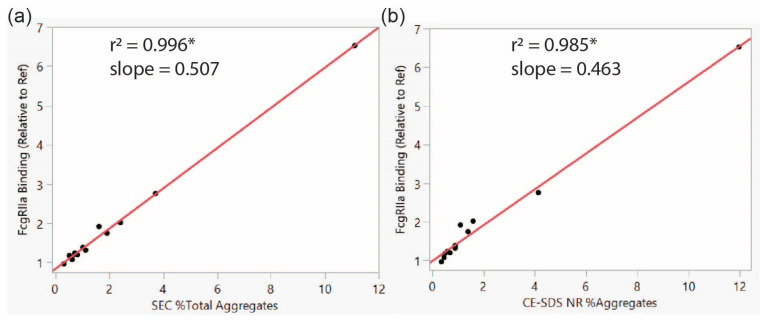
Correlation analysis for FcγRIIa 131R binding by SPR to % aggregate by SEC (**a**) and non-reduced CE-SDS (**b**) for data from Table 1 and Table 2. * Signifies significant *p*-value of <0.0001.

The forcibly degraded and photostability samples were examined for their physical attributes to determine the source of the increased binding to FcγRIIa compared to Ref. There was a relatively high correlation between FcγRIIa binding and oxidation level for several of the oxidizable amino acids (r^2^ 0.55–0.78) at three different methionine residues (252, 358, and 428) (Supplemental Figure S1). It is known that mAb oxidation can induce aggregation [42,46,47]. There was an even greater correlation between FcγRIIa binding and aggregate level. Aggregates, as determined by SEC and non-reduced capillary electrophoresis (NR CE-SDS), were found to have a significant correlation with FcγRIIa binding (Figure 1). Greater FcγRIIa binding was associated with increases in non-reducible aggregates (R CE-SDS), for the most part. However, there were two forcibly degraded samples (pH 8.0 and Cu^2+^) with levels of non-reducible aggregates similar to the photostability sample that did not show a similar increase in FcγRIIa binding. In summary, for this binding method format, FcγRIIa binding demonstrated a high correlation with aggregate levels, with 1% increases in mAb1 aggregation leading to approximately 50% more FcγRIIa binding.

### 3.2. Activity of Isolated Aggregate mAb Fractions

**Figure 2:**
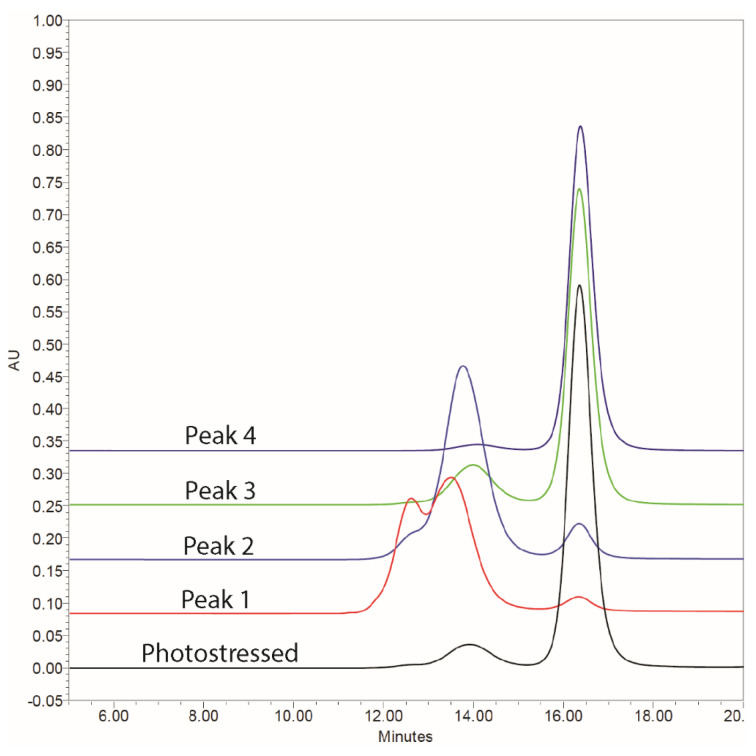
Analytical SEC of photostability sample (110% ICH) and fractions isolated from preparative SEC.

To further examine the impact of mAb1 aggregation on Fcγ receptor activity, mAb1 (batch C at 150 mg/mL) was exposed to a light stress of 110% of that outlined in ICH Q1B. This sample (110% ICH) was fractionated with preparative SEC. The aggregate and fragment levels, as well as the purity of the pooled fractions, were examined with analytical SEC and CE-SDS (reduced and non-reduced), as shown in Table 3 and Figure 2.

Peak 1, eluting between 500 and 570 mL by preparative size exclusion chromatography (Supplemental Figure S2), had the largest percentage of total aggregates, with 23% higher order aggregate. Peak 2 consisted of primarily dimer with similar levels of non-reducible aggregates to Peak 1. Peak 3 had a similar profile to the 110% ICH starting material. Peak 4 consisted of primarily monomer with a slightly lower purity than Ref with ~6% impurities from aggregates and fragments.

The 110% ICH light stressed sample and subsequent SEC fractions were analyzed for FcγRIIa, FcγRIIIa, and antigen binding by SPR compared to a Ref standard curve. The FcγRIIa assay data in Table 1 utilized biotinylated FcγRIIa bound to a Streptavidin chip, whereas the FcγRIIIa assay data in Table 1 was with FcγRIIIa covalently attached to a CM5 chip. To account for possible differences in Fcγ receptor orientation and binding capacity, the 110% ICH sample and isolated fractions were examined for FcγRIIa and FcγRIIIa binding, utilizing similarly prepared surfaces (biotin captured FcγR on Streptavidin surface) (Table 4).

Compared to Ref, FcγRIIa binding was considerably greater for the 110% ICH sample and isolated peaks than FcγRIIIa. Peak 4, with only 1.6% more aggregate compared to Ref, displayed higher binding to FcγRIIa, but not FcγRIIIa. There was a high correlation between % dimer (SEC) to FcγRIIa binding, indicating a 40% increase in binding with each 1% increase in mAb1 dimer for this assay format (Supplemental Figure S3). The correlation with % dimer (SEC) only suggested about a 10% increase in FcγRIIIa binding with every 1% increase in dimer. Antigen binding did not increase with aggregate levels, and decreased for Peaks 1 and 2 compared to Ref. The decrease in antigen binding was most likely related to the levels of non-reducible aggregate levels in Peaks 1 and 2, as determined by reduced CE-SDS. Perhaps the non-reducible aggregates were in a configuration less accessible to antigen binding.

### 3.3. Increased Fc Receptor Binding by SPR Is Avidity-Driven

Multiple groups have reported aggregate-driven increases in Fcγ receptor binding for immunoglobulins utilizing SPR [27,29,30]. It was hypothesized that the increase in Fcγ receptor binding by SPR was from the avidity-based binding of mAb aggregates to Fcγ receptors on the chip surface. Geuijen et al. showed that by reversing the format and presenting the mAb on the surface, the binding affinities of SPRi did not correlate with the presence of aggregates [30]. As sensorgrams were not provided for the mAb-down format and affinity values were not obtained with the FcγR-down format, it is difficult to compare the impact of the two setups. The hypothesis that increased binding to FcγR was avidity-driven was tested with FcγRIIa 131R, utilizing two different SPR affinity assay formats, mAb-down (non-avidity design) and Fc-receptor-down (avidity design), as shown in the schematic in Supplemental Figure S4. Initial studies utilized a ProA capture method for the mAb-down format. However, the captured amounts of mAb1 for Peak 1 and Peak 2 by ProA were markedly reduced. Since antigen binding was not substantially impacted by the 110% ICH light stress conditions, and the binding interaction of antigen and mAb1 is sufficiently stable to ensure consistent amounts of captured mAb during the association and dissociation phase with FcγRIIa, an antigen capture method was utilized instead. Ref and Peak 2 were tested with the two different binding formats. Ref, with minimal aggregation, was utilized as a negative control. Peak 2 was chosen because it contained substantial aggregation, mostly consisting of mAb1 dimer and monomer.

**Figure 3:**
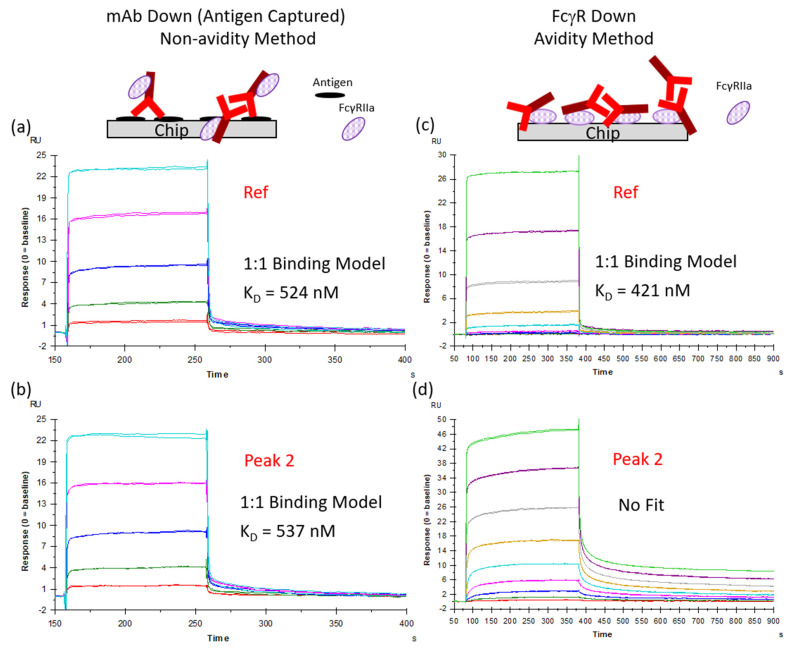
Increased FcγRIIa binding by SPR with mAb1 dimer is avidity-driven. FcγRIIa (131R) binding with mAb1 reference standard (Ref) and Peak 2 were measured in two different SPR formats: mAb-down (**a**,**b**) and FcγR-down (**c**,**d**). (**a**–**c**) Fitted to a steady-state affinity model. An appropriate model was not available to fit the bivalent and heterogeneous Peak 2 (**d**). Embedded schematics show simplified orientations on the SPR chip between the methods; see Supplemental Figures S4 and S5 for more detail. FcgRIIa concentrations for (**a**) and (**b**) were 6000, 2000, 667, 222, 74, and 25 nM. mAb1 concentrations for (**c**) and (**d**) were 1000, 333, 111, 37, 12, 4.1, 1.4, 0.5, and 0.2 nM.

Ref and Peak 2 were fitted to a steady-state affinity model for the mAb-down method (non-avidity-based), since the on- and off-rates were too fast for a binding kinetics model (see Supplemental Figure S5 for fitted data). Ref and Peak 2 had similar K_D_ values of 0.5 µM (Figure 3a,b), consistent with previous results for IgG1 and FcγRIIa [48]. Additionally, the responses for Ref and Peak 2 were similar in the non-avidity method (~23 RU).

However, as shown in Figure 3c,d, there were noticeable differences between Ref and Peak 2 with the avidity-based method (Fcγ receptor-down). Ref was fitted with the steady-state model and exhibited a K_D_ similar to results obtained with the antibody-down method. The slightly lower K_D_ value for the Ref with the Fc-receptor-down method (Figure 3c) fell outside the variability of the K_D_ values obtained with the mAb-down method for Ref and Peak 2 (Supplemental Table S1), and is most likely related to the fit for Ref not reaching an optimal plateau, as accomplished with the mAb-down method. There was no appropriate model available to fit Peak 2. The bivalent analyte model would only be appropriate if Peak 2 contained only the dimer, as opposed to a mixture. Peak 2 displayed off-rates visibly slower than Ref. Slower off-rates are usually seen when avidity is present. The binding response for Peak 2 was nearly twice that of Ref (47 vs. 28 RU). This is due to the increased size of the dimer in Peak 2 compared to monomeric Ref on the SPR signal. The response for Peak 2 was not exactly twice that of Ref, as Peak 2 was a mixture of a monomer and dimer.

### 3.4. Aggregation Increases FcγRIIa Activity More than Other Fc Receptors in Various Assay Formats

**Figure 4:**
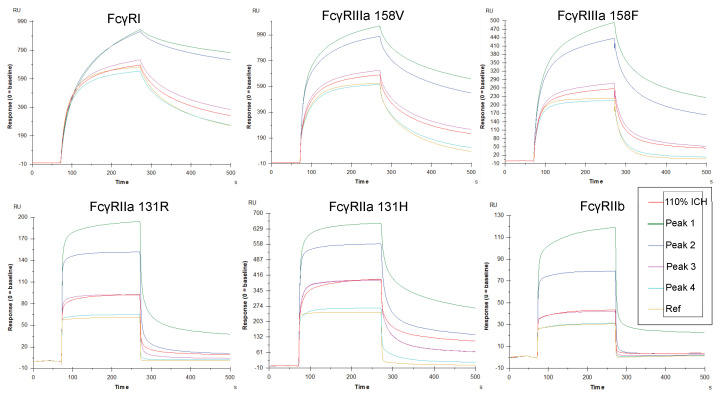
Reference-subtracted binding sensorgrams of photostability sample (110% ICH) and SEC-isolated fractions from SPR. Fc receptors were covalently immobilized to CM5 chips and 0.6 µM (90 µg/mL) mAb samples were injected over the surface.

**Figure 5:**
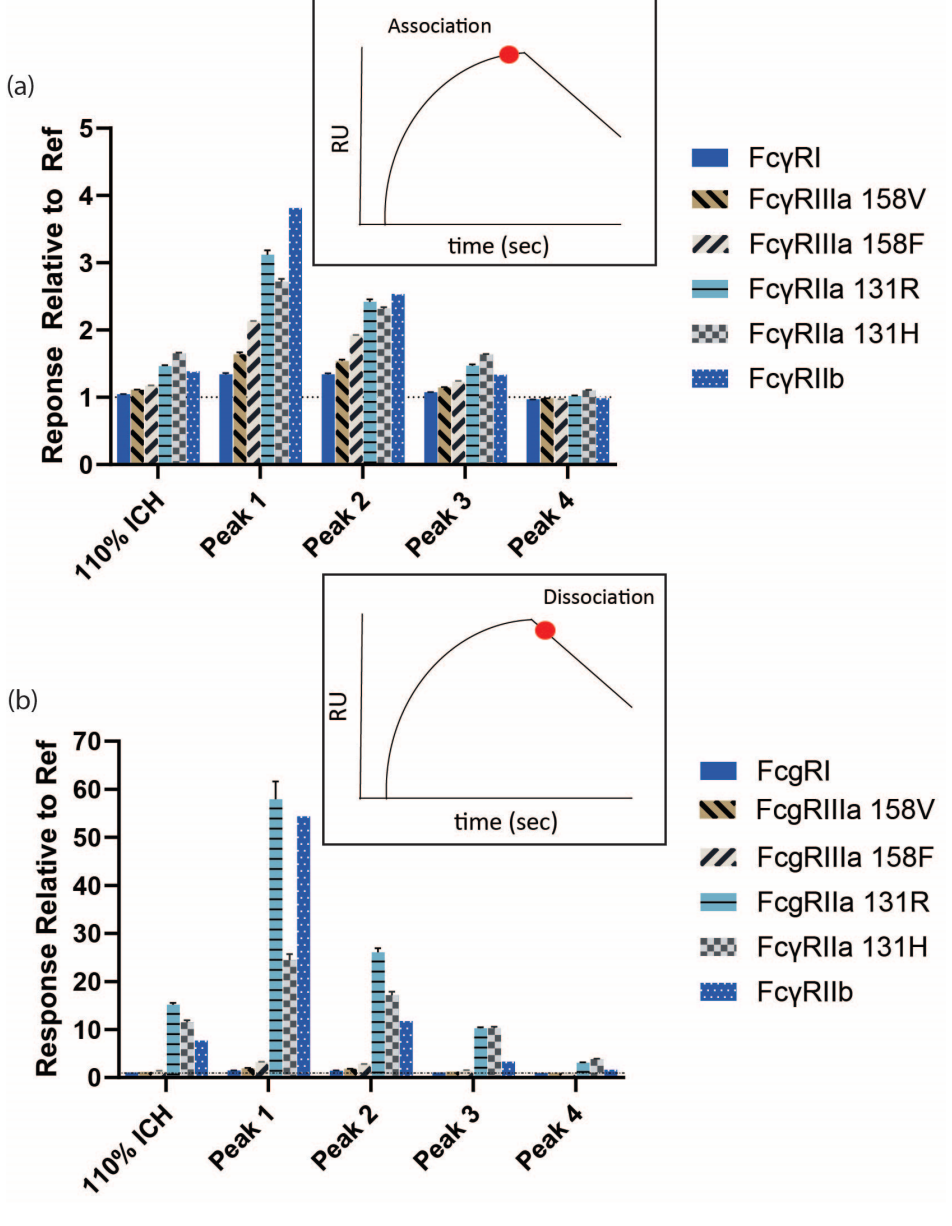
Binding responses of photostability sample (110% ICH) and SEC-isolated fractions from sensorgrams in Figure 4. Sample responses were divided by the Ref response. (**a**) Association phase. (**b**) Dissociation phase. The dotted line at the Fold Untreated Ref value of 1 indicates no change from Ref. Error bars represent standard deviation (*n* = 4).

The impact of mAb aggregation on the activity for multiple Fc receptors was evaluated in several assay formats, utilizing the 110% ICH mAb1 sample and subsequent SEC-isolated fractions. Responses during binding and dissociation were assessed by SPR by injecting mAb samples over covalently immobilized receptors (the SPR format enabling avidity). The binding and retention of mAb1 increased for all the Fcγ receptors and correlated with increasing levels of mAb1 aggregation. Relative to the Ref, the 110% ICH sample and Peaks 1–3 had greater binding responses for FcγRII (FcγRIIa 131R, FcγRIIa 131H, FcγRIIb) compared to FcγRI and FcγRIIIa (158V and 158F) (Figure 4 and Figure 5a, Supplemental Table S2). The amount of remaining mAb bound to the Fc receptors after the injection for all the samples compared to Ref was substantially higher for the FcγRII receptors compared to FcγRI and FcγRIII (Figure 4 and Figure 5b). Peak 4, with only 1.6% more aggregated mAb compared to Ref, displayed increased retention to FcγRII, but not FcγRI and FcγRIII. Although the increases in binding and retention for Peaks 1 and 2 for FcγRII appear less noticeable compared to sensorgrams for FcγRI and FcγRIII, the change in binding profiles for Peaks 1 and 2 for FcγRII compared to the Ref is striking. Ref binds FcγRII with weak affinity, and binding to FcγRII appears to require avidity-based binding and retention more than FcγRI and FcγRIII.

**Figure 6:**
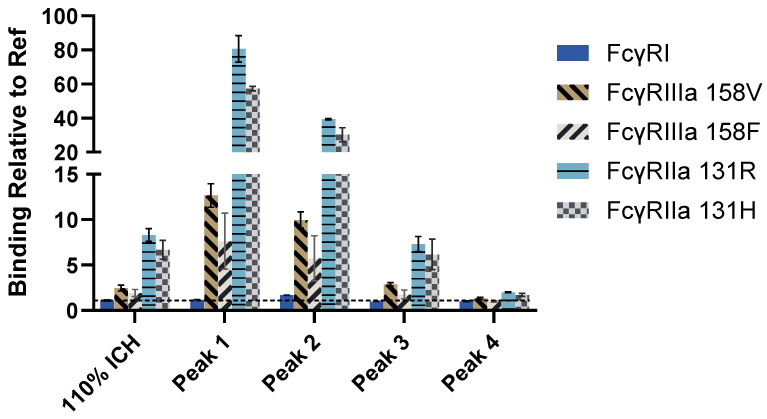
Solution-based competitive binding for photostability sample (110% ICH) and SEC-isolated fractions. The binding responses were calculated relative to the reference standard. The dotted line at the Binding Relative to Ref value of 1 indicates no change from Ref. Error bars represent standard deviation (*n* = 2).

Binding of mAb1 110% ICH and isolated fractions to Fc receptors were also examined with a solution-based competitive binding assay (see Supplemental Figure S6 for a schematic of the assay format). Binding was calculated relative to Ref (Figure 6 and Supplemental Figures S7–S11).

The relative binding for the low-affinity Fc receptors (FcγRIIa 131R, FcγRIIa 131H, FcγRIIIa 158V, and FcγRIIIa 158F) with the solution-based binding method correlated with the level of mAb1 aggregation. The relative binding for FcγRI was 1.7-fold greater for Peak 2 (the fraction with the greatest level of dimeric species (70%)) compared to Ref. It is interesting that Peak 1, with the higher level of total aggregates, only led to a 1.2-fold increase in relative binding for FcγRI, which did not correlate with mAb1 total aggregation levels and binding by avidity-based SPR. It could be that FcγRI is unable to bind multimeric mAb in solution as efficiently. Lux et al. found that increased valency did not lead to increased binding to FcγRI-expressing cells, as it did for FcγRII [25]. Additionally, cytokine signaling is thought to increase FcγRI affinity to immune complexes through clustering in lipid rafts or confirmational changes [49]. For all samples tested, the binding relative to Ref was greater for FcγRIIa (FcγRIIa 131R and FcγRIIa 131H) compared to FcγRI and FcγRIIIa (158V and 158F). The substantial increase in binding to FcγRII could be due to the presence of FcγRII dimers [50], which could lead to large complexes with mAb1 dimer. Another explanation could simply be that FcγRII binding is more dependent on avidity compared to the other FcγRs. In this assay format, c-terminally biotinylated Fcγ receptors are captured on Streptavidin, which contains four biotin-binding sites and could increase the possibility of avidity-based interactions. In any case, there is a clear increase in binding to mAb1 aggregates for FcγRIIa in solution compared to the other receptors.

**Figure 7:**
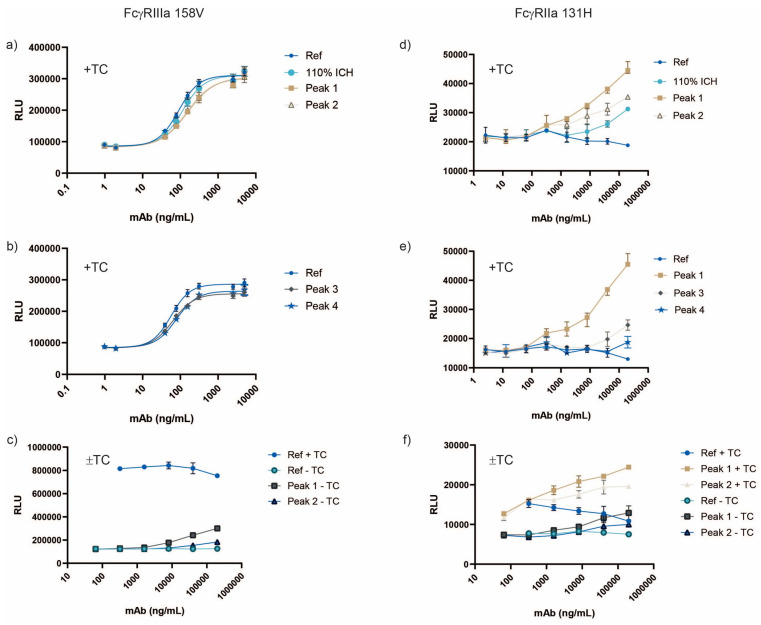
Reporter cell assays for photostability sample (110% ICH) and SEC-isolated fractions. Reporter cells expressing luciferase and FcγRIIIa 158V (**a**–**c**) or FcγRIIa 131R (**d**–**f**) were added to target cells (+TC) and mAb samples. Reporter cell activation was also evaluated with mAb samples with (+TC) and without target cells (−TC) (**c**,**f**). RLU = relative luminescence units.

**Figure 8:**
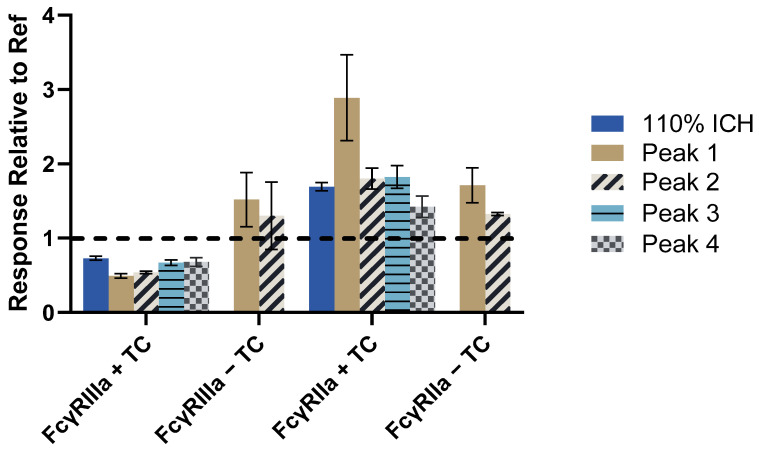
Summary of cell-based assay results presented in Figure 7. Cell-based reporter assay results for 110% ICH sample and SEC-isolated peaks. FcγRIIIa results with target cells were calculated based on relative potency values compared to Ref (*n* = 3 plates). The average RLU response relative to Ref at 200 µg/mL was calculated for FcγRIIIa without target cells and FcγRIIa (±target cells) (*n* = 2–12 replicates). The dotted line at the Response Relative to Ref value of 1 indicates no change from Ref. Error bars represent standard deviation.

Antibody-dependent cellular cytotoxicity (ADCC) for mAbs is often assessed with a surrogate reporter gene assay utilizing cells expressing FcγRIII in the presence of target cells [41]. Upon immune complex formation (target cell–mAb–FcγRIII reporter cell) and the initiation of downstream signaling, the reporter is expressed and the activation of FcγR can be quantified with a plate reader. The 110% ICH and isolated fractions were examined with reporter assays specific for FcγRIIIa (158V) and FcγRIIa (131H), which expressed luciferase when gene transcription by the nuclear factor of activated T-cells (NFAT) occurred after engagement with the FcγRs [41]. The FcγRIIIa reporter assay in the presence of target cells showed decreased activity for all samples compared to Ref (Figure 7a,b and Figure 8, Supplementary Table S3).

It was suspected that the 30% decrease in activity for the FcγRIIIa reporter assay with target cells was from an increase of ~1% fucose in the samples compared to Ref (Table 5). A change of 1% fucosylation has been shown to impact FcγRIII cell-based activity by ~20% [41]. The decrease in FcγRIII cell-based activity for the 110% ICH sample and isolated peaks compared to Ref could also be due to chemical modifications, since they were all similarly impacted in canonical methionine sites 252 and 428, which are known to reduce FcRn binding [37,43,44,45]. Modifications in the FcRn binding site by mutation or oxidation have been reported to impact FcγR activity [45,51,52,53]. Peak 4, with minimal aggregation and fragmentation but similar levels of methionine oxidation to the other fractions, did not show a decrease in binding to the Fcγ receptors or antigen compared to Ref (Table 4, Figure 5 and Figure 6). However, because of the slight increase in aggregation for Peak 4 compared to Ref, any decrease in binding activity to FcγR could potentially be masked by the aggregation with the avidity-based binding formats.

FcγRIIIa binding to Ref and Peak 4 with the non-avidity SPR method was utilized to test the hypothesis that oxidation led to reduced binding to FcγRIIIa, thereby reducing activity in the FcγRIIIa cell-based assay. Ref and Peak 4 were captured with the mAb1 antigen, and different concentrations of FcγRIIIa (158V) were injected over the captured Ref and Peak 4. There was no decrease in binding to FcγRIIIa for Peak 4 compared to Ref with the mAb-down method (Supplemental Figure S12). Thus, it is unlikely that the oxidation of methionine sites 252, 358, and 428 were causing the 30% reduction in the FcγRIIIa reporter assay with target cells for the 110% ICH sample and isolated peaks, and were in fact driven by increased fucosylation compared to Ref. The additional 20% decrease in activity for Peaks 1 and 2 in the cell-based FcγRIIIa reporter assay with target cells was likely due to the decrease in antigen binding (Table 4).

The activation of FcγRIIIa reporter cells alone, without target cells, was greater for Peaks 1 and 2 compared to Ref (Figure 7c and Figure 8) and agrees with results from Tada et al. [24], where aggregated mAbs activated effector cells without target cells.

Interestingly, the mAb1 standard did not show any activity in the FcγRIIa reporter assay in the presence of target cells with the conditions tested, whereas FcγRIIa activity increased for the 110% ICH sample and isolated fractions (Figure 7d,e and Figure 8). The activation of FcγRIIa reporter cells was the greatest with Peak 1 (+target cells). As opposed to FcγRIIIa, FcγRIIa appeared to be activated only in the presence of mAb1 aggregation, but increases were modest compared to the binding data. The FcγRIIa reporter assay system was verified to work in our hands with the anti-CD20 positive control (Promega) and CD20-expressing target cells, indicating that mAb1 is not as effective at activating FcγRIIa in the cell-based assay system compared to anti-CD20.

Overall, increased binding to aggregated mAb1 was found for all the Fcγ receptors for the avidity-based SPR assays, as well as the solution-based binding assay. FcγRII showed the greatest increase in binding for aggregated mAb1 with these methods. Results for these assays were all relative to Ref, which has a higher affinity for FcγRI and FcγRIIIa. There was a dramatic increase in binding for aggregated mAb1 compared to Ref for FcγRII. For the cell-based FcγR reporter gene assays, FcγRIIa activity increased only slightly in the presence of aggregates, while FcγRIIIa activity decreased.

## 4. Discussion

Utilizing mAb1 enriched for aggregates, we evaluated the impact of IgG1 aggregation on FcγR engagement in a variety of assay formats, including various SPR assay designs. Compared to the other Fcγ receptors, FcγRII was more sensitive to aggregates with binding and dissociation to mAb1 impacted even with samples containing a modest increase in aggregates compared to the corresponding reference standard (Peak 4). There was a clear difference in behavior for different immobilization formats with SPR (mAb-down vs. Fcγ receptor-down) definitively proving that, with SPR, the increase in binding from aggregated mAb is avidity-driven. The aggregation of mAb1 impacted the off-rate in the avidity-based SPR format (FcγR-down). It is important to use the correct model for SPR (e.g., bivalent analyte) to obtain accurate binding constants. Several studies have reported the affinity of the interaction of aggregated IgG with immobilized FcγRs utilizing 1:1 binding models [29,32]. A 1:1 binding model will not accurately describe the interaction of a mixture with one of the components (e.g., dimer) driving the binding and dissociation significantly more than the other (e.g., monomer). Similarly, steady-state measurements which capture only the responses during the association phase will not capture the contribution of the reduced off-rates driven from aggregates. In our case, there was no appropriate model to fit Peak 2 with the FcγR-down model, as Peak 2 was a heterogenous mixture with two binding sites. An appropriate model would take into account both the mixture concentrations and the two-step binding reaction for the dimer.

Three different avidity-based SPR assays were utilized to evaluate the impact of mAb1 aggregation on FcγRIIa 131R binding for Peak 2. The binding assay with C-terminal biotinylated FcγRIIa captured onto a Streptavidin (SA) chip utilizing a Ref standard curve (Assay 1) showed Peak 2 with a 28.6-fold increase in FcγRIIa binding compared to Ref (Table 4). The increase in binding for Peak 2 with the amine-coupled FcγRIIa (Assay 2) was only 2.3-fold greater than Ref (Figure 5a). Peak 2 was able to bind biotinylated FcγRIIa 12-fold more than amine-coupled FcγRIIa. The direct coupling of FcγRII receptors can decrease the activity of the receptor as well as lead to randomly oriented receptor on the chip. Both assay formats utilized high immobilization levels (1570 RU for the SA chip and 1958 RU for the CM5 chip), the same flow rate, and the same injection time, with RU values for mAb1 binding acquired 7 s before the end of the injection. The 12-fold increase in the binding of Peak 2 in Assay 1 was likely due to the high amounts of active FcγRIIa on the surface, coupled with avidity-based binding. The binding reaction did not reach equilibrium for Assay 1 because the mAb1 aggregate did not dissociate from the surface during the injection, causing the response to increase over the course of the injection (Supplementary Figure S13). There may also have been mass-transport differences during the injection for the mAb1 aggregate compared to the Ref with Assay 1. (Note also the differences between FcγRIIa and FcγRIIIa binding sensorgrams with the Assay 1 format—Supplementary Figure S13 vs. Supplementary Figure S14). The kinetic measurement with only 10 RU of biotinylated FcγRIIa on the surface (Assay 3) showed a 1.8-fold increase in binding response compared to Ref at 7 s before the end of the injection (Figure 3c,d). The approximate two-fold increase in response was directly related to the molecular weight of the dimer being twice that of monomer. The binding responses for all three formats were reference-subtracted with a blank flow cell upstream of the FcγRIIa flow cell. Reference subtraction removed non-specific bulk responses during the injection (no non-specific binding to the reference flow cell was present in the sensorgrams after injection for the mAb1 concentrations tested, and hence the responses in the blank flow cell were due to the bulk response during the injection).

The concentrations of all mAb1 samples were determined at 280 nm, utilizing the extinction coefficient for mAb1. The structure and composition of the aggregates in the samples were unknown. The extinction coefficient for aggregated mAb1, potentially making up a complex mixture of aggregates in various configurations, could differ for each aggregate type. This scenario would lead to inaccurate concentration results. Results from most binding and cell-based assays are concentration-dependent. Consequently, utilizing a correct concentration is critical for accurate results. The responses for Ref and Peak 2 in Figure 3a,b are the same, indicating that the concentration utilized for diluting Peak 2 was accurate. For all other mAb1 samples containing aggregates, the accuracy of the concentrations utilized in the binding and cell-based assays are unknown. However, the direct binding assay (Figure 4 and Figure 5) utilized the same dilution factor for each sample tested across the six different Fcγ receptors. Thus, the results across the different Fcγ receptors were not dependent on accurate concentrations and displayed increased binding to FcγRII over FcγRI and FcγRIIIa, in agreement with the rest of the avidity-based binding assay formats.

Few studies have examined the impact of aggregates on FcγR activity utilizing both binding and cell-based assay formats. Most of these utilized PBMCs or THP-1 cells containing two or more FcγRs. Only one of these reports utilized cells expressing a specific FcγR (FcγRIIa) [31], and it was found that with the concentrations tested the cell-based assay expressing FcγRIIa did not demonstrate increased activity with mAb aggregates, as found in the binding assay. To our knowledge, no reports thus far have examined the impact of mAb aggregates on binding, as well as cell-based activity utilizing cell lines specifically expressing FcγRIIa and FcγRIIIa.

The starting concentrations of mAb1 utilized for the FcγRIIIa reporter assay with target cells were 40-fold lower than those utilized in the FcγRIIIa reporter assay without target cells, indicating a greater efficiency in FcγRIIIa activation from receptor clustering with target engagement. IgG binding is necessary but not sufficient to activate FcγRs on effector cells, since physical receptor cross-linking is required for receptor triggering [3,54]. FcγRIIIa binding to mAbs is inhibited by fucosylation [55,56] and most likely limits the clustering or tight packing of effector cells with mAbs on target cells. Contrary to the FcγRIIIa binding data and the response with FcγRIIIa reporter cells alone, mAb1 aggregation did not increase FcγRIIIa reporter activation in the reporter assay with target cells. In other words, increased FcγRIIIa reporter activation occurred in the presence of target cells and was sensitive to mAb1 fucosylation levels rather than aggregation.

We found that mAb1 aggregates were required to elicit a response with the FcγRIIa reporter cells, but the activation was markedly reduced compared to the FcγRIIIa cell-based reporter assay. The differences in the behaviors of the two FcγRs could be due to structural differences between the binding interactions with mAb1 in the presence of target cells. The binding interfaces between IgG-Fc and both FcγRIIa and FcγRIIIa were structurally similar [57], apart from the N162 oligosaccharide expressed on FcγRIIIa, which is critical to antibody binding [56,58]. As the anti-CD20 positive control activated FcγRIIa-expressing reporter cells in the presence of CD20-expressing target cells, it is likely that mAb1 is unable to accommodate efficient interactions in the FcγRIIa cell-based assay in the presence of antigen-expressing target cells through clustering. This could be due to prohibitive geometry preventing the formation of the target cell–mAb–reporter cell clustering, which was seen previously with different mAbs targeting different sites on CD20 [59]. The increased levels of mAb1 aggregates may have increased the activation on FcγRIIa reporter cells by creating immune complexes promoting avidity, not through clustering with multivalent binding sites on the target cells. It would be interesting to understand the impact of aggregation to cell-based FcγRIIa reporter assays, utilizing mAbs with greater activity in that assay system.

Aggregation is a critical quality attribute monitored and controlled for therapeutic mAbs before batches can be released for use. The results of this study show that obtaining relative binding results with effector-competent mAb samples containing aggregate levels above the reference standard should be approached with caution, especially for FcγRII. Interpreting FcγR results in studies geared towards understanding FcγR biology should also take into account the impact of differing levels of aggregation for different samples.

## Figures and Tables

**Table 1:** Binding and purity results for photostability samples. Batch A was at 150 mg/mL and batch B at 35 mg/mL. FcγR and FcRn binding by SPR compared to a Ref standard curve. Samples were exposed per ICH Q1B guidelines (light) or wrapped in foil (dark). Ref = reference standard; Agg = aggregates; NR = non-reduced; Non-Red = non-reducible; R = reduced.

Condition	Binding Relative to Ref			% Total Agg (SEC)	% Total Agg (NR CE-SDS)	% Non-Red Agg (R CE-SDS)	% Fragments (R CE-SDS)
	FcγRIIa 131R	FcγRIIIa 158V	FcRn				
Ref	1.0	1.0	1.0	0.5	0.2	0.7	1.4
Batch A—Light	6.5	1.1	0.9	11.2	12.0	6.2	1.4
Batch A—Dark	1.1	1.0	1.0	0.6	0.5	0.7	1.4
Batch B—Light	2.8	0.9	0.9	3.7	4.1	5.1	1.5
Batch B—Dark	1.0	0.9	1.0	0.3	0.4	0.6	1.4

**Table 2:** Binding and purity results for forcibly degraded samples. Forcibly degraded batch B mAb1 samples were analyzed for FcγRIIa (131R) binding by SPR compared to a Ref standard curve. Aggregate levels were determined by SEC. Aggregate levels and fragment levels were determined by CE-SDS. Agg = aggregates; NR = non-reduced; Non-Red = non-reducible; R = reduced.

Condition	FcγRIIa Binding *	% Total Agg (SEC)	% Total Agg (NR CE-SDS)	% Non-Red Agg (R CE-SDS)	% Fragments (R CE-SDS)
Ref	1.0	0.5	0.2	0.7	1.4
Temp Only	1.2	0.7	0.6	1.1	2.5
pH 4.0	1.2	0.5	0.5	0.7	3.7
pH 8.0	1.8	1.9	1.4	5.6	3.1
10 ppm H_2_O_2_	1.2	0.8	0.7	1.1	2.5
20 ppm Fe^3+^	1.9	1.6	1.1	1.4	2.9
20 ppm Cu^2+^	1.4	1.0	0.9	6.2	2.5
200 mM Glucose	1.3	1.1	0.9	1.6	16.8
1 ppm H_2_O_2_	1.2	0.7	0.7	1.1	2.6
0.5 ppm Fe^3+^	2.0	2.4	1.6	2.1	3.0

* Relative to Ref.

**Table 3:** Purity results of 110% ICH Q1B photostability mAb1 Batch C and SEC-isolated fractions. Agg = aggregates, H.O. = higher order; Red = reduced; Non-Red = non-reducible; NR = non-reduced.

Sample	SEC				% Total Agg (NR CE-SDS)	% Non-Red Agg (R CE-SDS)	% Fragments (R CE-SDS)
	% Monomer	% Dimer	% H.O. Agg	% Total Agg			
Ref	99.5	0.4	0.1	0.5	0.2	0.7	1.4
110% ICH	89.1	10.1	0.9	11	9.1	5.2	2.3
Peak 1	20.9	56.0	23.0	79.0	72.9	21	2.6
Peak 2	25.2	70.3	4.5	74.8	68.7	19.6	2.4
Peak 3	86.5	13.1	0.3	13.4	13.7	6.8	1.8
Peak 4	97.9	1.99	0.04	2.0	2.4	3.4	2.4

**Table 4:** Binding results of the photostability sample (110% ICH) and isolated SEC fractions. FcγRIIa (131R), FcγRIIIa (158V), and antigen binding by SPR compared to a Ref standard curve for mAb1 after 110% ICH Q1B treatment and fraction isolation by SEC. Biotinylated FcγRIIa and FcγRIIIa were attached to an SA chip and the antigen was covalently attached to a CM5 chip.

Sample	Binding (Relative to Ref)		
	FcγRIIa Binding	FcγRIIIa Binding	Antigen Binding
Ref	1.0	1.0	1.0
110% ICH	4.3	1.8	1.1
Peak 1	26.7	7.6	0.8
Peak 2	28.6	6.6	0.8
Peak 3	6.2	2.0	1.0
Peak 4	1.4	1.0	0.9

**Table 5:** LC-MS data for post-translational modifications of 110% ICH sample and isolated peaks. All values are percentages. M = methionine, N = asparagine.

Sample	%M252 Oxidation	%M358 Oxidation	%M428 Oxidation	%N325 Deamidation	% %Afucosylated
Ref	1.84	0.50	0.73	<1	6.1
110% ICH	24.45	4.26	17.82	<1	5.4
Peak 1	25.38	4.68	18.28	<1	5.1
Peak 2	25.81	4.37	17.94	<1	5.3
Peak 3	25.05	4.04	17.59	<1	4.9
Peak 4	23.85	4.12	17.09	<1	5.6

## Data Availability

The data from this study are available from the corresponding author (W.J.W.) upon reasonable request.

## Supplementary Materials

The following supporting information can be downloaded at https://www.mdpi.com/article/10.3390/antib14020031/s1, Figure S1: FcγRIIa binding and Fc oxidation; Figure S2: Preparative size exclusion chromatography for 110% ICH treated mAb1; Figure S3: FcγRIIa binding and % SEC dimer; Figure S4: A simplified schematic describing the differences of a) Fc-receptor down and b) mAb down SPR methods; Figure S5: SPR steady-state affinity fitted curves; Figure S6: Schematic for Lumit no wash competitive binding solution assay; Figure S7: Representative FcγRI no-wash competitive binding graph; Figure S8: Representative FcγRIIIa 158V no-wash competitive binding graph; Figure S9: Representative FcγRIIIa 158F no-wash competitive binding graph; Figure S10: Representative FcγRIIa 131H no-wash competitive binding graph; Figure S11: Representative FcγRIIa 131R no-wash competitive binding graph. Figure S12: FcγRIIIa (158V) binding to antigen captured mAb1 as measured by SPR; Figure S13: Sensorgrams for 110% ICH and isolated fractions binding to FcγRIIa (131R) data from Table 4; Figure S14: Sensorgrams for 110% ICH isolated fractions binding to FcγRIIIa (158V) data from Table 4; Table S1: Sensorgrams for 110% ICH isolated fractions binding to FcγRIIIa (158V) data from Table 4; Table S2: Binding responses as measured by receptor-down SPR method and no wash competitive binding solution assay; Table S3: Cell-based reporter assay results for 110% ICH sample and SEC isolated peaks.

## Abbreviations

The following abbreviations are used in this manuscript:

ADA	anti-drug antibody
ADCC	antibody-dependent cellular cytotoxicity
ADCP	antibody-dependent cellular phagocytosis
CE-SDS	capillary electrophoresis sodium dodecyl sulfate
FcRn	neonatal Fc receptor
mAb	monoclonal antibody
IVIG	intravenous immunoglobulin
PBMC	peripheral blood mononuclear cells
Ref	one mAb1 reference standard batch used throughout the study
RU	resonance units
RLU	relative luminescence units
SA	streptavidin
SEC	size exclusion chromatography
SPR	surface plasmon resonance
