# Supplementary material for: Impact of Monoclonal Antibody Aggregates on Effector Function Characterization
Source: Antibodies (Basel). 2025 Apr 2;14(2):31. doi: 10.3390/antib14020031 (PMC12015860; doi:10.3390/antib14020031)
Supplement: Supplementary file 1 [file antibodies-14-00031-s001.zip › antibodies-3457216-supplementary.pdf]

Supplementary Figures and Tables

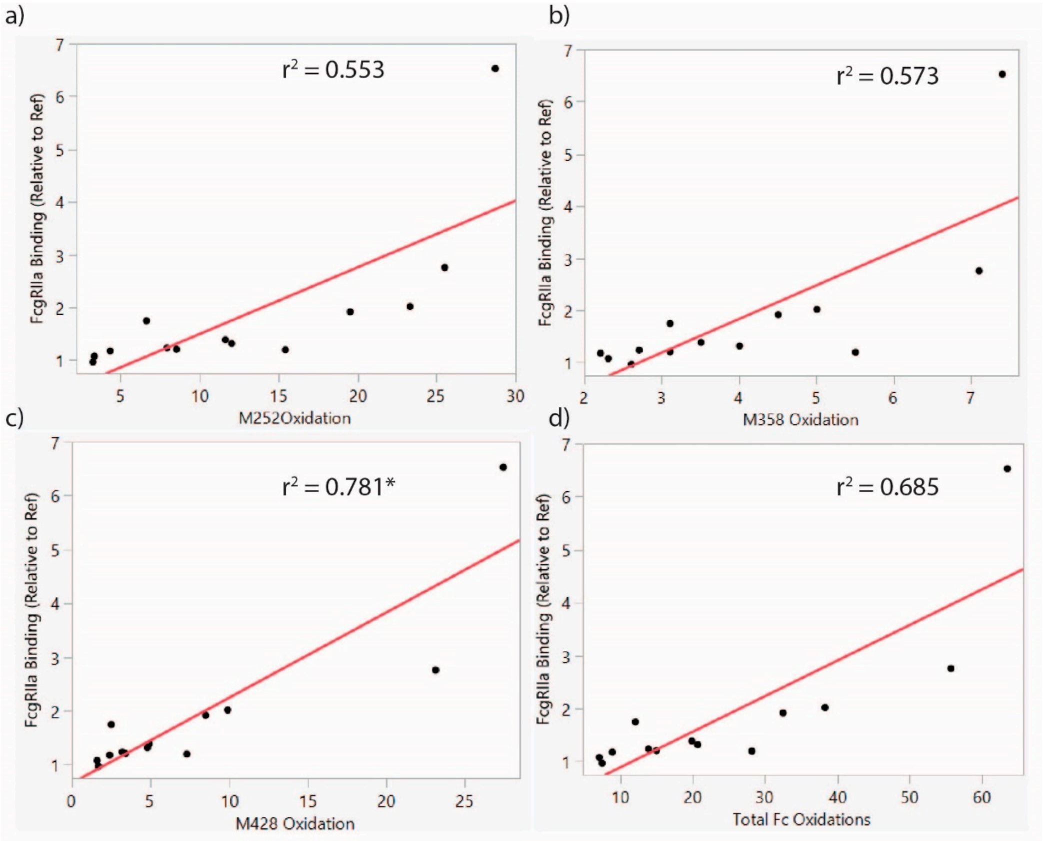

Figure S1: FcγRIIa binding and Fc oxidation. Correlation analysis for %FcγRIIa 131R binding by SPR to %oxidation in Fc methionine residues M252 (a); M358 (b); M428 (c), and the sum for the three M252, M358, M428 (d). All p-values were <0.05. *p-value <0.0001

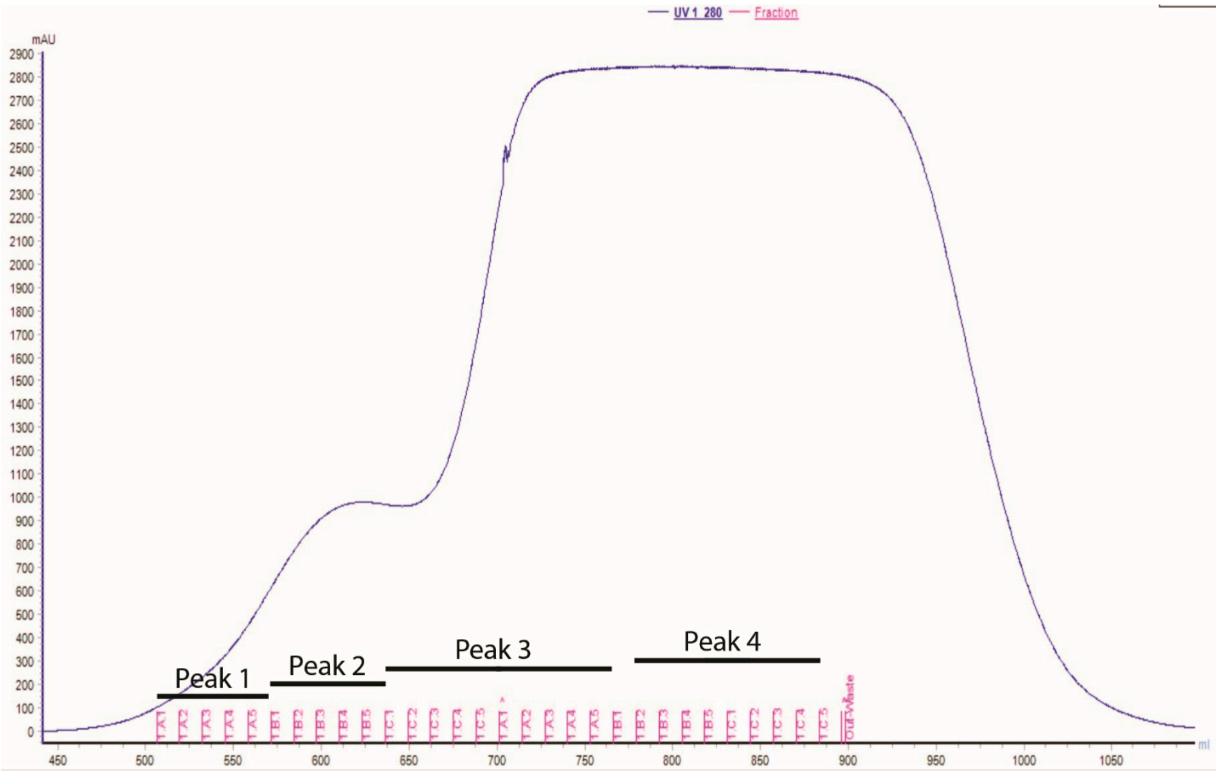

Figure S2: Preparative size exclusion chromatography for 110% ICH treated mAb1. Fractions for Peak 1,2,3,4 are shown.

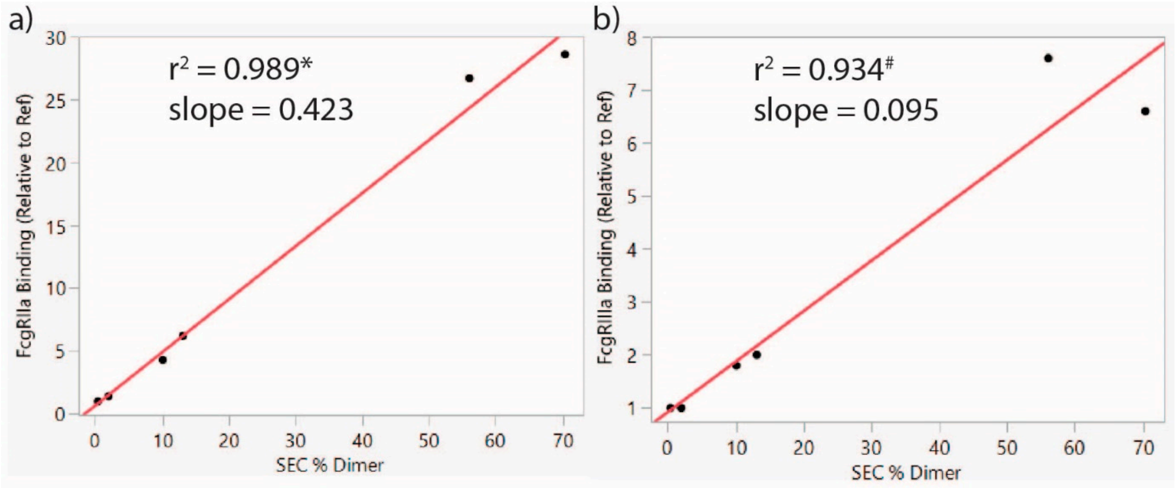

Figure S3: FcγRIIa binding and % SEC dimer. Correlation analysis for %FcγRIIa 131R (a) and FcgRIIIa 158V (b) binding to %SEC dimer. The 110% ICH treated mAb1 and isolated fractions were tested with SPR and results were compared to a Ref standard curve. Biotinylated FcgR was attached to a streptavidin chip. *p-value of <0.0001; #p-value of 0.0017.

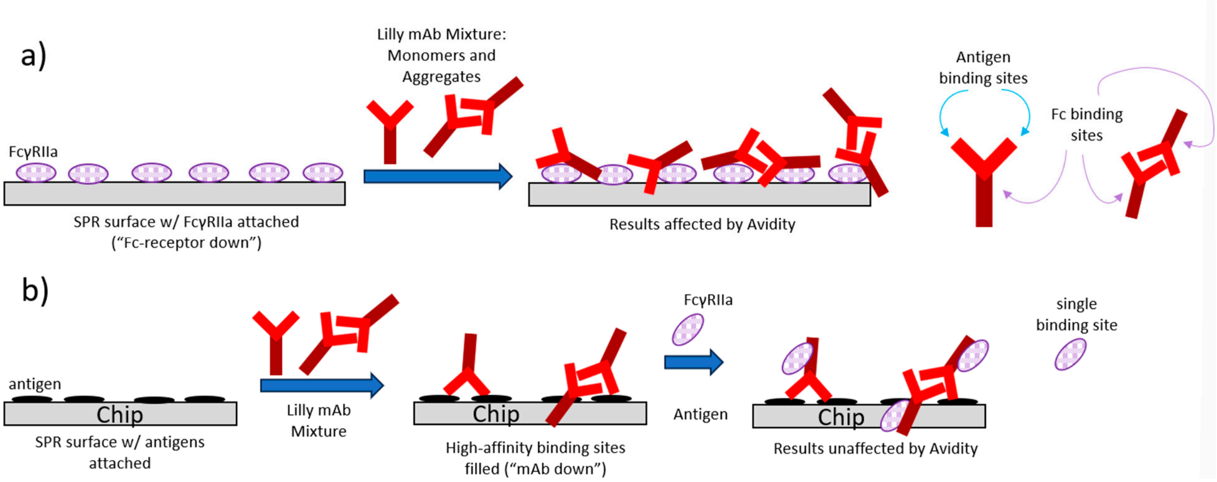

Figure S4: A simplified schematic describing the differences of a) Fc-receptor down and b) mAb down SPR methods. Dimeric species shown are examples of how the dimer may be formed. It is currently unknown how the mAb monomers are linked to each other to form dimer and higher order structures.

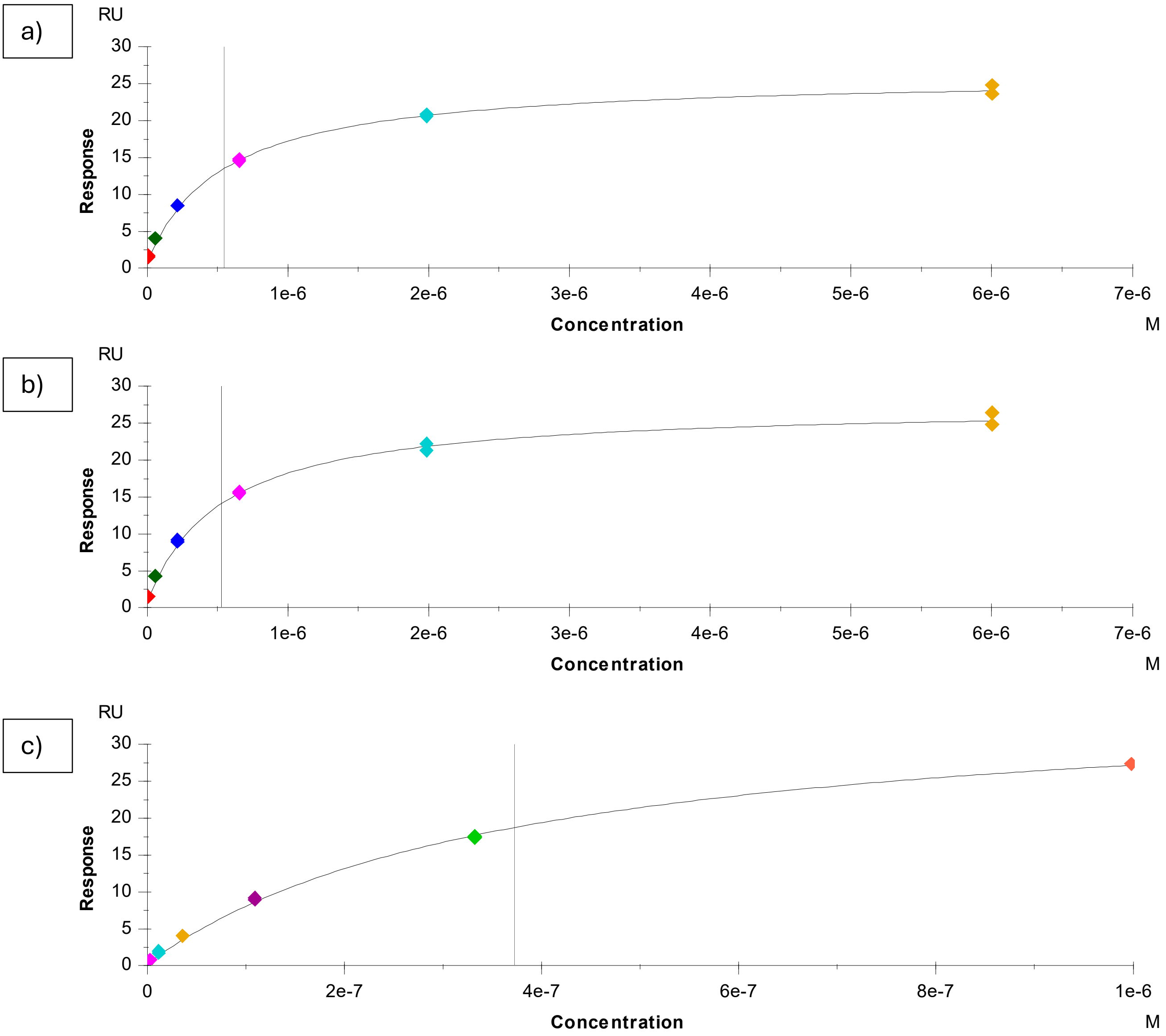

Figure S5: SPR steady-state affinity fitted curves. Steady-state affinity fits (1:1 model) for mAb Down (a, b) and Receptor Down (c) for Ref (a, c) and Peak 2 (b).

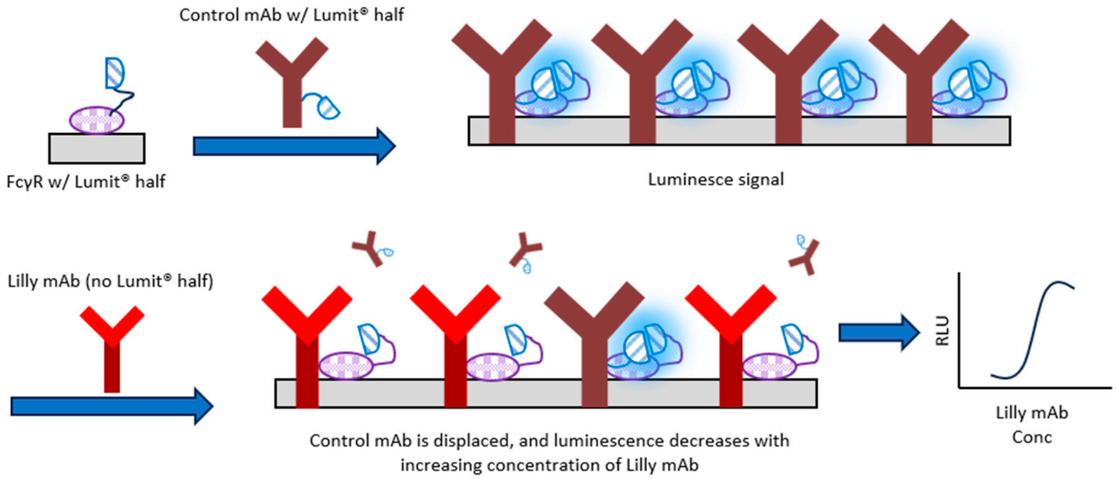

Figure S6: Schematic for Lumit® no wash competitive binding solution assay.

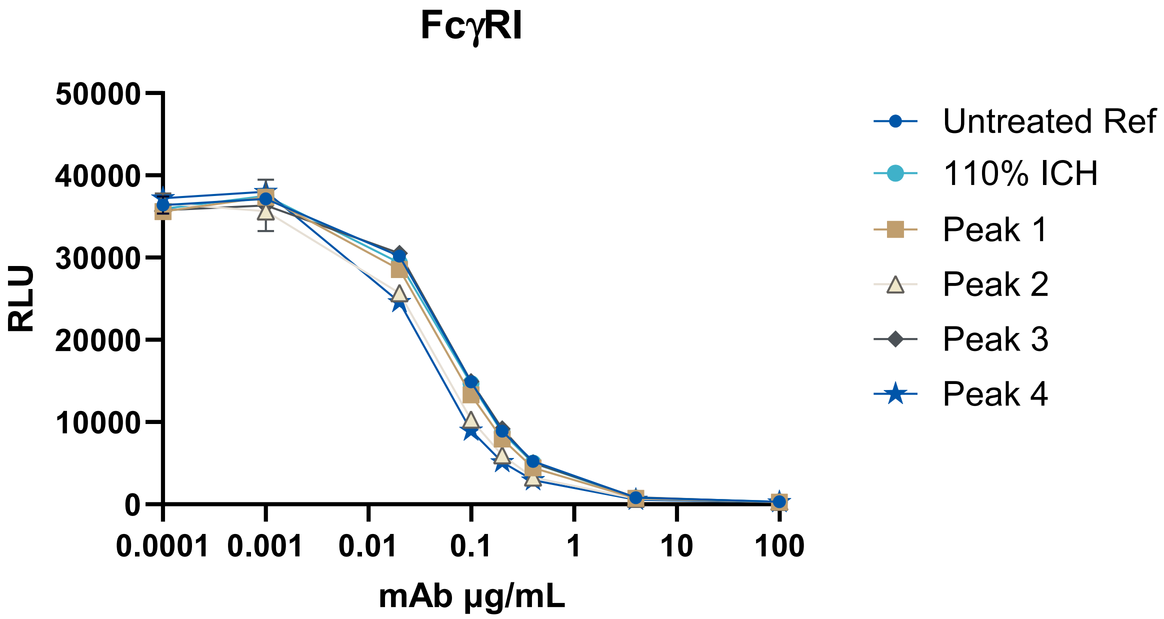

Figure S7: Representative FcgRI no-wash competitive binding graph. RLU = relative luminescence units.

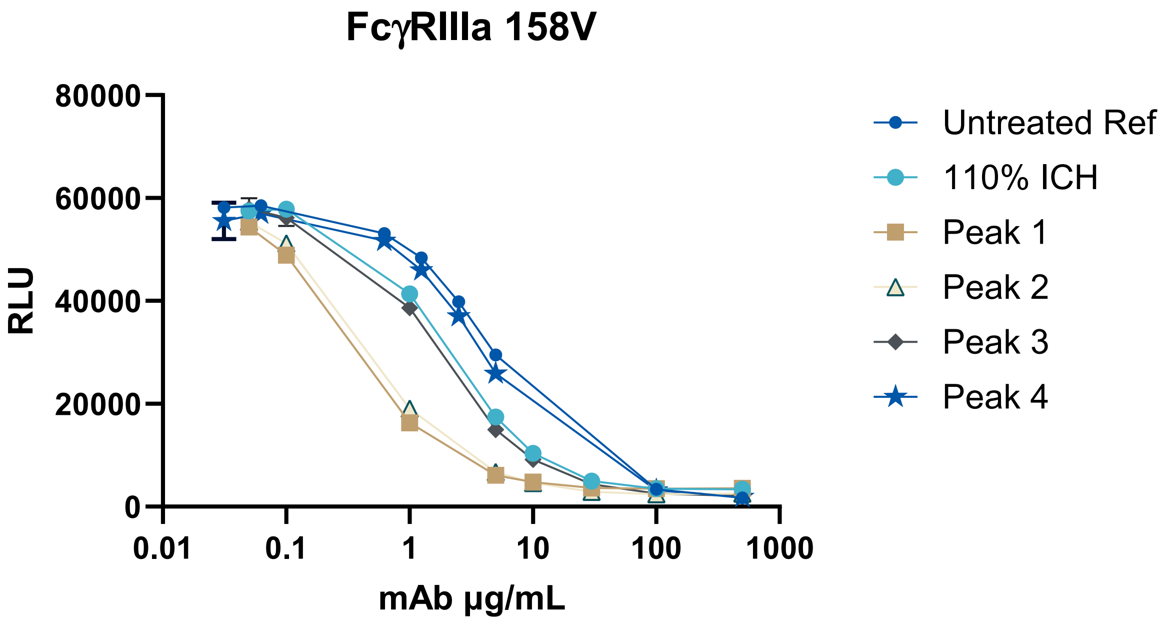

Figure S8: Representative FcgRIIIa 158V no-wash competitive binding graph. RLU = relative luminescence units.

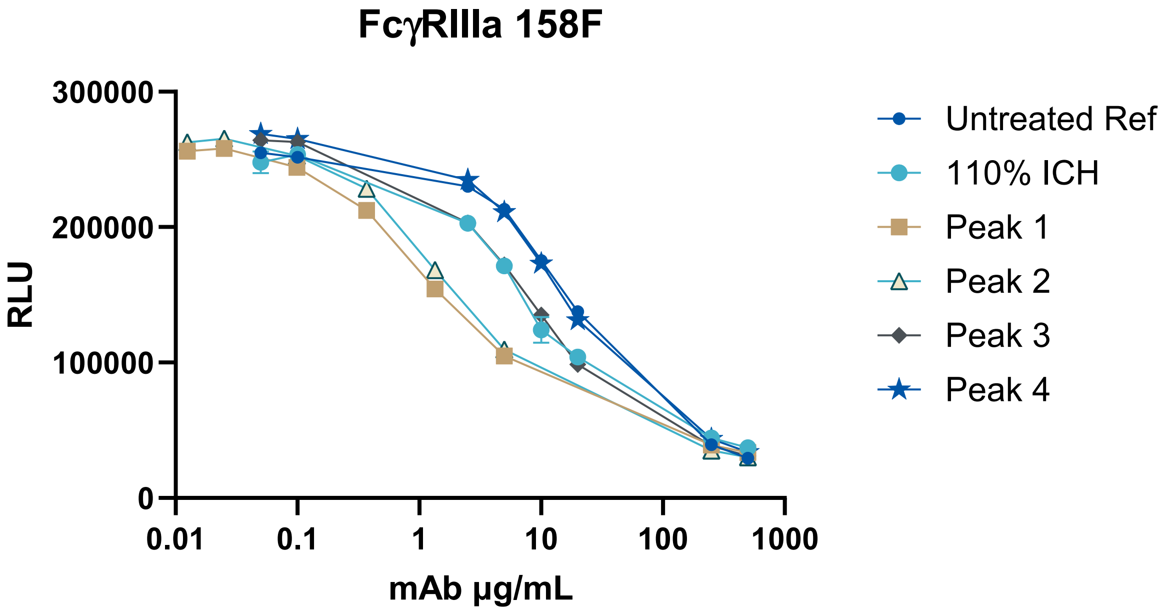

Figure S9: Representative FcgRIIIa 158F no-wash competitive binding graph. RLU = relative luminescence units.

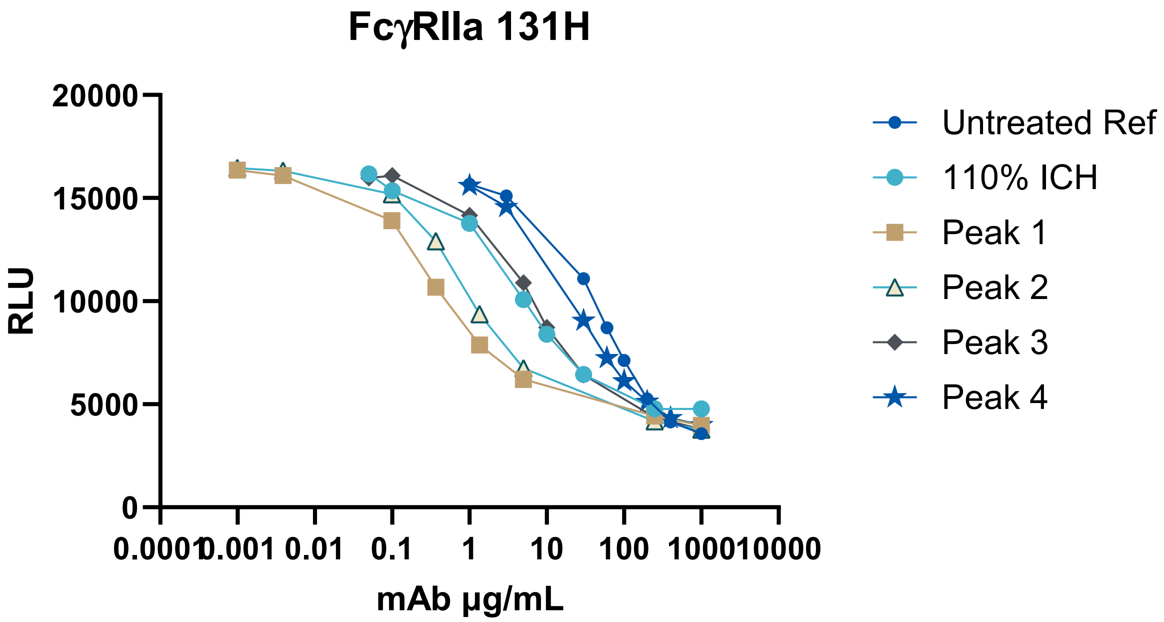

Figure S10. Representative FcgRIIa 131H no-wash competitive binding graph. RLU = relative luminescence units.

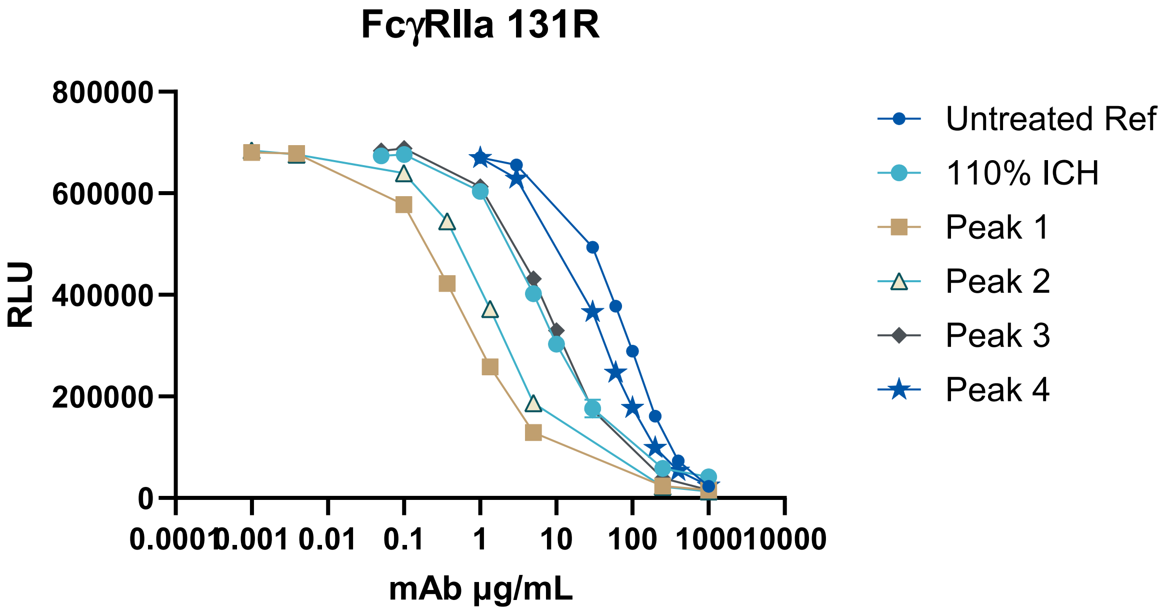

Figure S11: Representative FcgRIIa 131R no-wash competitive binding graph. RLU = relative luminescence units.

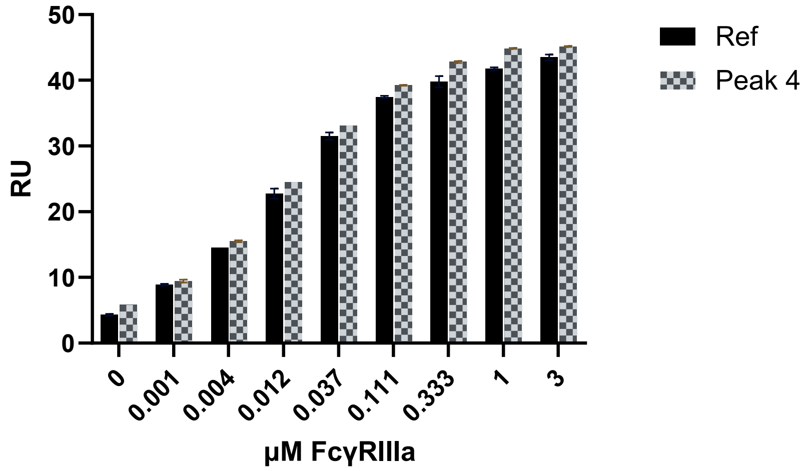

Figure S12: FcgRIIIa (158V) binding to antigen captured mAb1 as measured by SPR. The mAb down format was utilized to remove avidity. Error bars represent standard deviation (n=2).

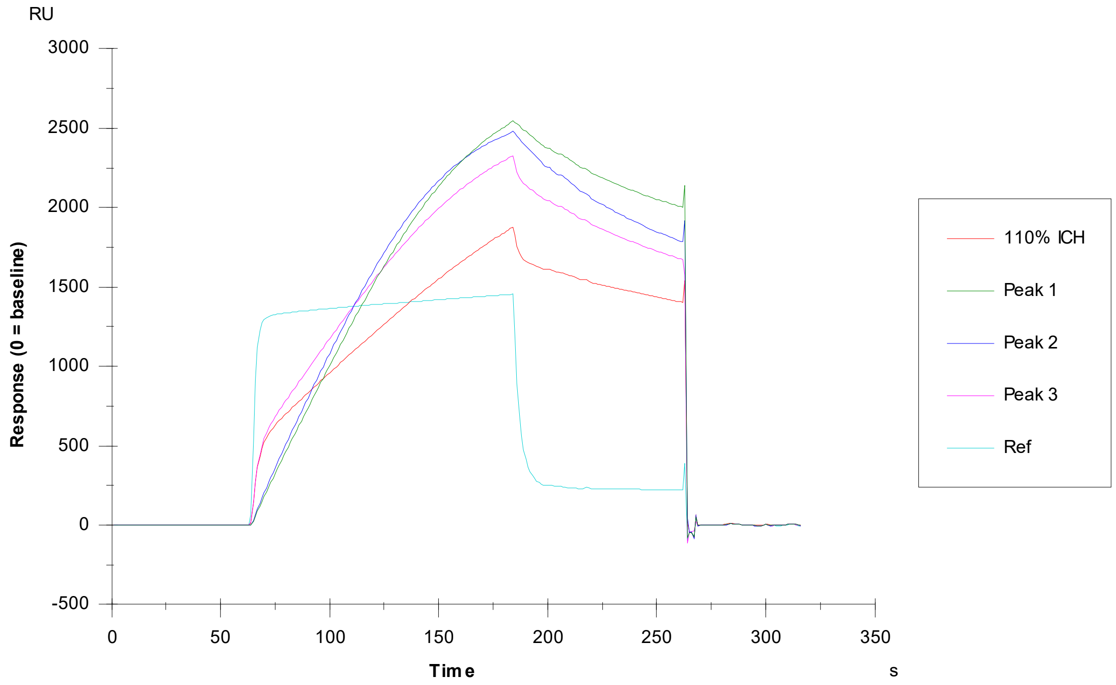

Figure S13: Sensorgrams for 110% ICH and isolated fractions binding to FcgRIIa (131R) data from Table 4. Biotinylated FcγRIIa was attached to an SA chip. Sensorgrams are shown for the highest concentration of mAb1 tested in the assay: Ref, 70 µg/mL; 110% ICH, 20 µg/mL; Peak 1, 5 µg/mL; Peak 2, 5 µg/mL; and Peak 3, 10 µg/mL.

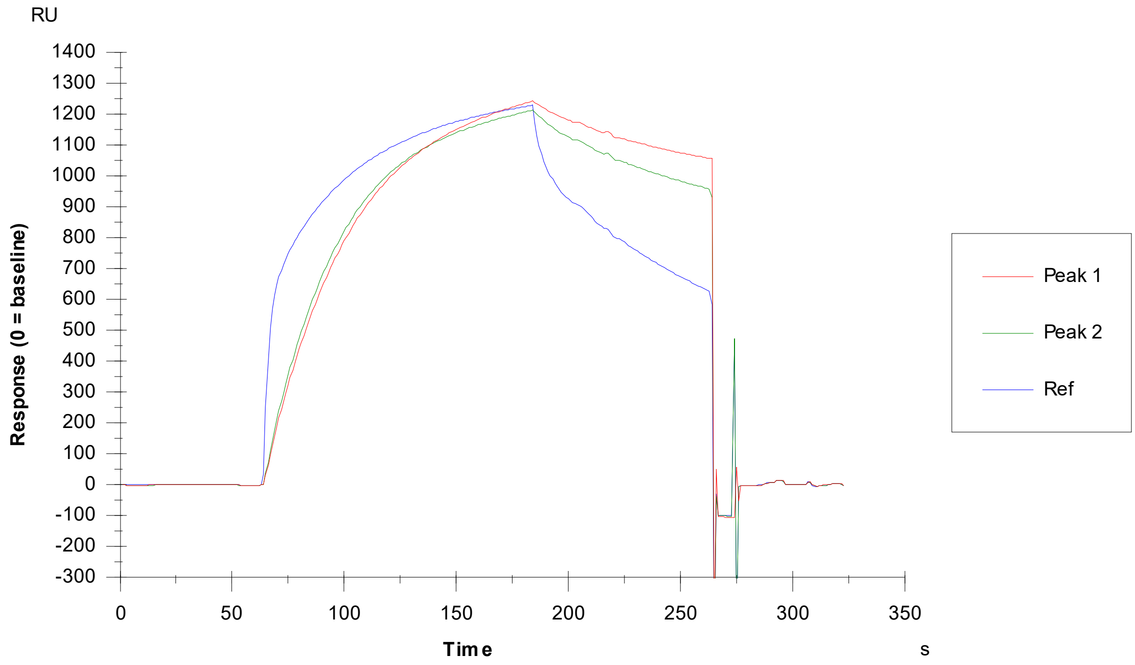

Figure S14: Sensorgrams for 110% ICH isolated fractions binding to FcgRIIIa (158V) data from Table 4. Biotinylated FcγRIIIa was attached to an SA chip. Sensorgrams are shown for the highest concentration of mAb1 tested in the assay: Ref, 70 µg/mL; Peak 1, 10 µg/mL; Peak 2, 10 µg/mL.

Table S1. Steady-state fit results from Figure 3. Results of steady-state affinity fits utilizing Biacore evaluation software for sensorgrams from Figure 3 (a,b,c). Standard deviations are shown in parenthesis (n=3).

**Fit**	**Assay Design**	**Sample**	**K_D (M)**
Steady-state affinity (1:1)	mAb Down (Non-Avidity Method)	Ref	5.2e-7 (±1.8e-8)
		Peak 2	5.4e-7 (±8.8e-9)
	Fc Receptor Down (Avidity Method)	Ref	4.2e-7 (±4.8e-8)

Table S2: Binding responses as measured by receptor-down SPR method and no wash competitive binding solution assay. The responses during the association or dissociation phase in the SPR sensorgrams for 110% ICH and isolated fractions were divided by the respective response for the untreated standard (Ref). Sample potencies were calculated in the no wash competitive binding assay relative to Ref and multiplied by 100.

Type of assay	**Fold-response (Relative to Ref) 0.6 μM SPR (Avidity) Association Phase**						**Fold-response (Relative to Ref) 0.6 μM SPR (Avidity) Dissociation Phase**						**Solution Based Potency (Relative to Ref)**				
**FcgR**	FcgRI	FcgRIIa 131R	FcgRIIa 131H	FcgRIIb	FcgRIIIa 158V	FcgRIIIa 158F	FcgRI	FcgRIIa 131R	FcgRIIa 131H	FcgRIIb	FcgRIIIa 158V	FcgRIIIa 158F	FcgRI	FcgRIIa 131R	FcgRIIa 131H	FcgRIIIa 158V	FcgRIIIa 158F
**Ref**	1.0	1.0	1.0	1.0	1.0	1.0	1.0	1.0	1.0	1.0	1.0	1.0	1.0	1.0	1.0	1.0	1.0
**110% ICH**	1.0	1.5	1.7	1.4	1.1	1.2	1.1	15.2	11.5	7.7	1.2	1.4	1.1	8.3	6.7	2.5	1.9
**Peak 1**	**1.3**	3.1	2.7	3.8	1.6	2.1	**1.5**	**58.0**	**24.4**	**54.4**	2.0	3.3	**1.2**	**80.6**	**57.3**	12.7	7.6
**Peak 2**	**1.3**	2.4	2.3	2.5	1.5	1.9	**1.5**	**26.1**	**17.1**	**11.8**	1.8	2.8	**1.7**	**39.3**	**30.3**	10.0	5.7
**Peak 3**	1.1	1.5	1.6	1.3	1.1	1.2	1.1	**10.3**	**10.3**	**3.4**	1.2	1.5	1.0	**7.3**	**6.2**	**2.9**	1.8
**Peak 4**	1.0	1.0	1.1	1.0	1.0	1.0	1.0	**3.1**	**3.9**	**1.6**	1.0	1.0	1.0	**2.0**	**1.7**	**1.3**	1.0

Table S3: Cell-based reporter assay results for 110% ICH sample and SEC isolated peaks. FcgRIIIa results with target cells were calculated based on relative potency values compared to reference standard (n=3). Average response relative to reference standard at 200 μg/mL shown for FcgRIIIa without target cells and FcgRIIa (± target cells) (n = 2-12).

**Sample**	**FcgRIIIa 158V %**	**FcγRIIIa 131H -Target Cells**	**FcgRIIa 131H +Target Cells**	**FcgRIIa 131H – Target Cells**
**110% ICH**	0.7	n.t.	1.7	n.t.
**Peak 1**	0.5	1.5	**2.9**	1.1
**Peak 2**	0.5	1.3	1.8	1.0
**Peak 3**	0.7	n.t.	1.8	n.t.
**Peak 4**	0.7	n.t.	1.4	n.t.

n.t. not tested
